# MVPA-Light: A Classification and Regression Toolbox for Multi-Dimensional Data

**DOI:** 10.3389/fnins.2020.00289

**Published:** 2020-06-04

**Authors:** Matthias S. Treder

**Affiliations:** School of Computer Science & Informatics, Cardiff University, Cardiff, United Kingdom

**Keywords:** machine learning, classification, decoding, regression, MVPA, regularization, cross-validation, toolbox

## Abstract

MVPA-Light is a MATLAB toolbox for multivariate pattern analysis (MVPA). It provides native implementations of a range of classifiers and regression models, using modern optimization algorithms. High-level functions allow for the multivariate analysis of multi-dimensional data, including generalization (e.g., time x time) and searchlight analysis. The toolbox performs cross-validation, hyperparameter tuning, and nested preprocessing. It computes various classification and regression metrics and establishes their statistical significance, is modular and easily extendable. Furthermore, it offers interfaces for LIBSVM and LIBLINEAR as well as an integration into the FieldTrip neuroimaging toolbox. After introducing MVPA-Light, example analyses of MEG and fMRI datasets, and benchmarking results on the classifiers and regression models are presented.

## 1. Introduction

Multivariate pattern analysis (MVPA) refers to a set of multivariate tools for the analysis of brain activity or structure. It draws on supervised learning, a branch of machine learning mainly dealing with classification and regression problems. Multivariate classification has been used in EEG-based brain-computer interfaces since at least the 1980s (Farwell and Donchin, 1988), but it did not become a mainstream tool in cognitive neuroscience until the late 2000s (Mur et al., 2009; Pereira et al., 2009; Blankertz et al., 2011; Lemm et al., 2011). MVPA was first popularized by the seminal work of Haxby et al. (Haxby et al., 2001; Norman et al., 2006; Haxby, 2012). In an fMRI study, the authors provided evidence that visual categories (such as faces and houses) are associated with distributed representations across multiple brain regions. MVPA is designed to exploit such multivariate patterns by taking into account multiple voxels or channels simultaneously. This constitutes a major difference between MVPA and traditional statistical methods such as *t*-test and analysis of variance (ANOVA). Traditional statistical tests are often univariate i.e., a test is performed for each dependent variable, for instance voxel or EEG channel, separately. In contrast to MVPA, such tests are blind to the distributed information encoded in the correlations between different spatial locations.

To highlight this difference with an example, consider a hypothetical visual experiment: In each trial, subjects are presented an image of either a face or a house and their brain activity is recorded using fMRI. To make sure that they maintain attention, subjects are instructed to indicate via a button press whether the image represents a face or a house. This experiment will be referred to as “faces vs. houses” throughout this paper. To investigate the difference between the brain responses to faces vs. houses, a *t*-test can be applied to answer the question “Is the activity *at a specific voxel* different for faces vs. houses?.” In contrast, MVPA addresses the more general question “Is the *pattern of brain activity* different for faces vs. houses?.” This example illustrates that univariate statistics and MVPA inhabit opposite ends of a spectrum between *sensitivity* (“Is there an effect?”) and *localizability* (“Where is the effect?”). A classical univariate test might be unable to detect a specific effect because it is blind to multivariate dependencies (low sensitivity) but any effect it does detect is perfectly localized to a single voxel. In contrast, MVPA gains statistical power by capitalizing on correlations between different locations (high sensitivity) but it is difficult to attribute an effect to a specific brain location (low localizability). A MVPA technique called *searchlight analysis* (see glossary) attempts to cover the middle ground between these two extremes. As this comparison illustrates, MVPA should be considered as a complement, rather than a competitor, to traditional statistical methods. Finally, there are other ways in which MVPA and traditional statistics differ. For instance, MVPA includes kernel methods that are sensitive to non-linear relationships and it makes extensive use of techniques such as *cross-validation* that control for *overfitting*.

To use MVPA as part of a neuroimaging analysis pipeline, numerous excellent MATLAB toolboxes have been developed over the years, including the Amsterdam Decoding and Modeling Toolbox (ADAM) (Fahrenfort et al., 2018), BCILAB (Kothe and Makeig, 2013), Berlin BCI toolbox (Blankertz et al., 2016), CoSMoMVPA (Oosterhof et al., 2016), Decision Decoding ToolBOX (DDTBOX) (Bode et al., 2019), Donders Machine Learning Toolbox (DMLT) (github.com/distrep/DMLT), Pattern Recognition for Neuroimaging Toolbox (PRoNTo) (Schrouff et al., 2013), and The Decoding Toolbox (TDT) (Hebart et al., 2015). Beyond MATLAB, the currently most popular computer languages for machine learning are Python and R, with outstanding toolboxes such as Scikit Learn (Pedregosa et al., 2011) for Python and Caret (Kuhn, 2008) and MLR (Bischl et al., 2000) for R. A comprehensive comparison of MVPA-Light with all of these toolboxes is beyond the scope of this paper, but what sets it apart is the adherence to all of the following design principles:

- *Self-contained*: unlike many toolboxes that provide wrappers for existing classifiers, the backbone of MVPA-Light is native implementations of various classifiers, regression models, and their corresponding optimization algorithms (Trust-Region Newton, Dual Coordinate Descent). As a result, MVPA-Light works out-of-the-box, without the need for additional toolboxes or code compilation.
- *Transparent*: the toolbox has a shallow code base with well-documented functions. In many cases, the function call stack has a depth of two within the toolbox. For instance, a call to mv_classify using an LDA classifier triggers calls to functions such as mv_check_inputs, train_lda, and test_lda. Although the train/test functions might call additional optimization functions, most of the work is done at these two shallowest levels. To preserve the shallowness, high-level functions replicate some code that might be shared otherwise. Object orientation and encapsulation is avoided in favor of the more transparent MATLAB structs.
- *Fast*: all models and high-level functions are written with speed as a prime concern. In some cases, the need for speed conflicts with the out-of-the-box requirement. For instance, Logistic Regression and SVM use iterative optimization algorithms written in MATLAB. However, these algorithms potentially run faster using compiled code. To this end, an interface is provided for LIBSVM (Chang et al., 2011) and LIBLINEAR (Fan et al., 2008), two C implementations of Logistic Regression and SVM for users who do not shy away from compiling the code on their platform.
- *Modular and pluggable*: it is possible, and intended, to harvest parts of the code such as the classifiers for other purposes. It is also easy to plug the toolbox into a larger neuroimaging analysis framework. An interface for FieldTrip (Oostenveld et al., 2011) is described in the Methods section.
- *High-level interface*: common MVPA tasks such as searchlight analysis and time generalization including cross-validation can be performed with a few lines of MATLAB code. Many of the hyperparameters required by classifiers and regression models are automatically selected by MVPA-Light, taking the burden of hyperparameter selection off the user.

It is worth noting that MVPA-Light is a purely statistical toolbox. That is, it assumes that data has been preprocessed with a neuroimaging toolbox and comes in the shape of MATLAB arrays. Many neuroimaging toolboxes (e.g., FieldTrip, SPM, EEGLAB) store the imaging data in such arrays, so that MVPA-Light can easily be used as a plugin tool. This comes with the perk that adaptation to different imaging modalities is straightforward.

### 1.1. MVPA Glossary

MVPA comes with its own set of commonly used terms, many of which are borrowed from machine learning. Since they are used extensively throughout the paper, a glossary is provided here. Fully understanding these concepts can be challenging so unfamiliar readers are referred to review papers on MVPA (Mur et al., 2009; Pereira et al., 2009; Misaki et al., 2010; Grootswagers et al., 2017; Varoquaux et al., 2017). For an in-depth introduction to machine learning refer to standard textbooks (Bishop, 2007; Hastie et al., 2009; James et al., 2013).

- *Binary classifier*. A classifier trained on data that contains two classes, such as in the “faces vs. houses” experiment. If there is more than two classes, the classifier is called a multi-class classifier.
- *Classification*. One of the primary applications of MVPA. In classification, a classifier takes a multivariate pattern of brain activity (referred to as *feature vector*) as input and maps it onto a categorical brain state or experimental condition (referred to as *class label*). In the “faces vs. houses” experiment, the classifier is used to investigate whether patterns of brain activity can discriminate between faces and houses.
- *Classifier*. An algorithm that performs classification, for instance Linear Discriminant Analysis (LDA) and Support Vector Machine (SVM).
- *Classifier output*. If a classifier receives a pattern of brain activity (feature vector) as input, its output is a predicted class label e.g., “face.” Many classifiers are also able to produce class probabilities (representing the probability that a brain pattern belongs to a specific class) or decision values.
- *Class label*. Categorical variable that represents a label for each sample/trial. In the “faces vs. houses” experiment, the class labels are “face” and “house.” Class labels are often encoded by numbers, e.g., “face” = 1 and “house” = 2, and arranged as a vector. For instance, the class label vector [1, 2, 1] indicates that a subject viewed a face in trial 1, a house in trial 2, and another face in trial 3.
- *Cross-validation*. To obtain a realistic estimate of classification or regression performance and control for overfitting, a model should be tested on an independent dataset that has not been used for training. In most neuroimaging experiments, there is only one dataset with a restricted number of trials. K-fold cross-validation makes efficient use of such data by splitting it into k different folds. In every iteration, one of the k folds is held out and used as test set, whereas all other folds are used for training. This is repeated until every fold served as test set once. Since cross-validation itself is stochastic due to the random assignment of samples to folds, it can be useful to repeat the cross-validation several times and average the results. See Lemm et al. (2011) and Varoquaux et al. (2017) for a discussion of cross-validation and potential pitfalls.
- *Data*. From the perspective of a classifier or regression model, a dataset is a collection of samples (e.g., trials in an experiment). Each sample consists of a brain pattern and a corresponding class label or response. In formal notation, each sample consists of a pair (**x**, *y*) where **x** is a feature vector and *y* is the corresponding class label or response.
- *Decision boundary*. Classifiers partition feature space into separate regions. Each region is assigned to a specific class. Classifiers make predictions for a test sample by looking up into which region it falls. The boundary between regions is known as decision boundary. For linear classifiers, the decision boundary is also known as a hyperplane.
- *Decision value*. Classifiers such as LDA and SVM produce decision values which can be thresholded to produce class labels. For linear classifiers and kernel classifiers, a decision value represents the distance to the decision boundary. The further away a test sample is from the decision boundary, the more confident the classifier is about it belonging to a particular class. Decision values are unitless.
- *Decoder*. An alternative term for a *classifier* or *regression model* that is popular in the neuroimaging literature. The term nicely captures the fact that it tries to invert the encoding process. In encoding e.g., a sensory experience such as viewing a face is translated into a pattern of brain activity. In decoding, one starts from a pattern of brain activity and tries to infer whether it was caused by a face or a house stimulus.
- *Feature*. A feature is a variable that is part of the input to a model. If the dataset is tabular with rows representing samples, it typically corresponds to one of the columns. In the “faces vs. houses” experiment, each voxel represents a feature.
- *Feature space*. Usually a real vector space that contains the feature vectors. The dimensionality of the feature space is equal to the number of features.
- *Feature vector*. For each sample, features are stored in a vector. For example, consider a EEG measurement with three electrodes Fz, Cz, and Oz and corresponding voltages 40, 65, and 97 μV. The voltage at each EEG sensor represents a feature, so the corresponding feature vector is the vector [40, 65, 97] ∈ ℝ^3^.
- *Fitting (a model)*. Same as *training*.
- *Hyperparameter*. A parameter of a model that needs to be specified by the user, such as the type and amount of regularization applied, the type of kernel, and the kernel width γ for Gaussian kernels. From the user's perspective, hyperparameters can be nuisance parameters: it is sometimes not clear a priori how to set them, but their exact value can have a substantial effect on the performance of the model.
- *Hyperparameter tuning*. If it is unclear how a hyperparameter should be set, multiple candidate values can be tested. Typically, this is done via nested cross-validation: the training set is again split into separate folds. A model is trained for each of the candidate values and its performance is evaluated on the held-out fold, called validation set. Only the model with the best performance is then taken forward to the test set.
- *Hyperplane*. For linear classifiers, the decision boundary is a hyperplane. In the special case of a two-dimensional feature space, a hyperplane corresponds to a straight line. In three dimensions, it corresponds to a plane.
- *Loss function*. A function that is used for training. The model parameters are optimized such that the loss function attains a minimum value. For instance, in Linear Regression the sum of squares of the residuals serves as a loss function.
- *Metric*. A quantitative measure of the performance of a model on a test set. For example, precision/recall for classification or mean squared error for regression.
- *Model*. In the context of this paper, a model is a classifier or regression model.
- *Multi-class classifier*. A classifier trained on data that contains three or more classes. For instance, assume that in the “faces vs. houses” experiment additional images have been presented depicting “animals” and “tools.” This would define four classes in total, hence classification would require a multi-class classifier.
- *Overfitting*. Occurs when a model over-adapts to the training data. As a consequence, it will perform well on the training set but badly on the test set. Generally speaking, overfitting is more likely to occur if the number of features is larger than the number of samples, and more likely for complex non-linear models than for linear models. Regularization can serve as an antidote to overfitting.
- *Parameters*. Models are governed by parameters e.g., beta coefficients in Linear Regression or the weight vector **w** and bias *b* in a linear classifier.
- *Regression*. One of the primary applications of MVPA (together with classification). Regression is very similar to classification, but it aims to predict a continuous variable rather than a class label. For instance, in the ‘faces vs. houses' experiment, assume that the reaction time of the button press has been recorded, too. To investigate the question “Does the pattern of brain activity in each trial predict reaction time?,” regression can be performed using reaction time as responses.
- *Regression model*. An algorithm that performs regression, for instance Ridge Regression and Support Vector Regression (SVR).
- *Regularization*. A set of techniques that aim to reduce overfitting. Regularization is often directly incorporated into training by adding a penalty term to the loss function. For instance, L1 and L2 penalty terms are popular regularization techniques. They reduce overfitting by preventing coefficients from taking on too large values.
- *Response*. In regression, responses act as the target values that a model tries to predict. They play the same role that class labels play in classification. Unlike class labels, responses are continuous e.g., reaction time.
- *Searchlight analysis*. In neuroimaging analysis, a question such as “Does brain activity differentiate between faces and houses?” is usually less interesting than the question “Which brain regions differentiate between faces and houses?.” In other words, the goal of MVPA is to establish the presence of an effect *and* localize it in space or time. Searchlight analysis intends to marry statistical sensitivity with localizability. It is a well-established technique in the fMRI literature, where a searchlight is defined e.g., as a sphere of 1 cm radius, centered on a voxel in the brain (Kriegeskorte et al., 2006). All voxels within the radius serve as features for a classification or regression analysis. The result of the analysis is assigned to the central voxel. If the analysis is repeated for all voxel positions, the resultant 3D map of classification accuracies can be overlayed on a brain image. Brain regions that have discriminative information then light up as peaks in the map. Searchlight analysis is not limited to spatial coordinates. The same idea can be applied to other dimensions such as time points and frequencies.
- *Testing*. The process of applying a trained model to the test set. The performance of the model can then be quantified using a metric.
- *Test set*. Part of the data designated for testing. Like with training sets, test sets are automatically defined in cross-validation, or they can arise naturally in multi-site studies or in experiments with different phases.
- *Training*. The process of optimizing the parameters of a model using a training set.
- *Training set*. Part of the data designated for training. In cross-validation, a dataset is automatically split into training and test sets. In other cases, a training set may arise naturally. For instance, in experiments with different phases (e.g., memory encoding and memory retrieval) one phase may serve as training set and the other phase as test set. Another example is multi-site studies, where a model can be trained on data from one site and tested on data from another site.
- *Underfitting*. Occurs when a classifier or regression model is too simple to explain the data. For example, imagine a dataset wherein the optimal decision boundary is a circle, with samples of class 1 being inside the circle and samples of class 2 outside. A linear classifier is not able to represent a circular decision boundary, hence it will be unable to adequately solve the task. Underfitting can be checked by fitting a complex model (e.g., kernel SVM) to data. If the complex model performs much better than a more simple linear model (e.g., LDA) then it is likely that the simple model underfits the data. In most neuroimaging datasets, overfitting is more of a concern than underfitting.

The rest of the paper is structured as follows. The high-level functions of the toolbox are described, followed by an introduction of the classifiers and regression models. Then, example analyses are presented using a publicly available Wakeman and Henson (2014, 2015) MEEG dataset and the Haxby et al. (2001) fMRI dataset. Finally, a benchmarking analysis is conducted wherein the computational efficiency of the classifiers and regression models in MVPA-Light is compared to models in other toolboxes in MATLAB, Python, and R.

## 2. Materials and Methods

### 2.1. Requirements

A standard desktop computer is sufficient to run MVPA-Light. The RAM requirement is dictated by the memory footprint of the dataset. Since some functions operate on a copy of the data, it is recommended that the available RAM exceeds the size of the dataset by at least a factor of two (e.g., 4+ GB RAM for a 2 GB dataset). MVPA-Light is supported by MATLAB 2012a and more recent versions. The Statistics toolbox is required at some points in the toolbox (e.g., for calculating *t*-values). The cluster permutation test in mv_statistics uses the Image Processing toolbox to extract the clusters.

### 2.2. Getting Started

MVPA-Light is shipped with a set of example scripts (in the /examples subfolder) and an example EEG dataset. These scripts cover both the high-level functions in MVPA-Light and calling the train/test functions manually. The best starting point is to work through the example scripts and then adapt them to one's purpose. An up-to-date introduction to the toolbox with relevant hyperlinks is provided on the GitHub page (github.com/treder/mvpa-light).

The EEG data has been taken from the BNCI-Horizon-2020 repository (http://bnci-horizon-2020.eu/database). It consists of three mat files corresponding to three subjects (subject codes VPaak, VPaan, and VPgcc) from the auditory oddball paradigm introduced in Treder et al. (2014). Out of the experimental conditions, the “SynthPop” condition has been selected. Attended and unattended deviants are coded as class 1 and 2. The 64 EEG channels in the original dataset have been reduced to 32 channels.

To give a concrete code example, consider the “faces vs. houses” experiment. For each trial, the BOLD response has been recorded for all voxels. This yields a [samples x voxels] data matrix for one subject, where the samples correspond to trials and the voxels serve as features. The matrix is denoted as X. Each trial corresponds to either a “face” or a “house” stimulus. This is encoded in a vector of class labels, denoted as clabel, that contains 1's and 2's (“face” = 1, “house” = 2). Then the following piece of code performs 10-fold cross-validation with 2 repetitions. LDA is used as classifier and area under the ROC curve (AUC) is calculated as a classification metric.


cfg = [];
cfg.model       = 'lda';
cfg.metric      = 'auc';
cfg.cv          = 'kfold';
cfg.k           = 10;
cfg.repeat      = 2;



auc = mv_classify(cfg, X, clabel);


The output value auc contains the classifier performance measure, in this case a single AUC value averaged across test folds and repetitions. mv_classify is part of the high-level interface that will be discussed next.

### 2.3. High-level Interface

**Figure 1:**
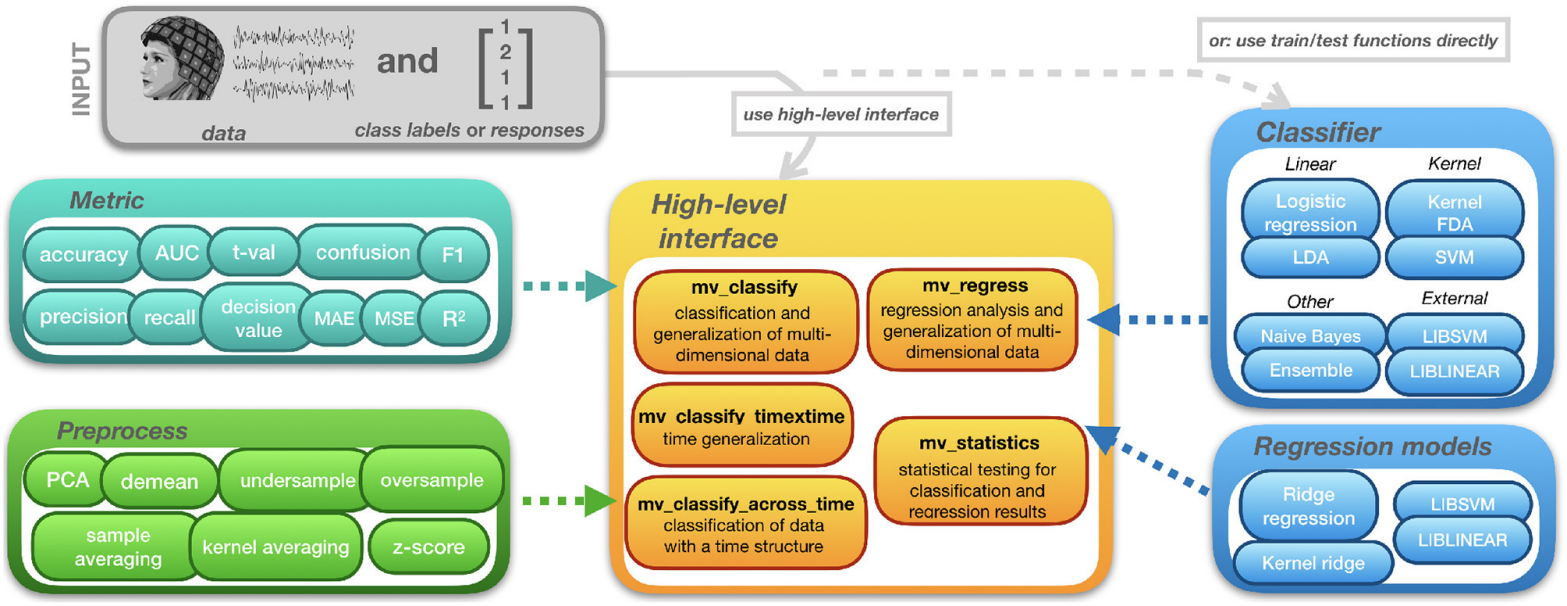
Structure of MVPA-Light.

The structure of MVPA-Light is depicted in Figure 1. The toolbox can be interacted with through high-level functions that cover common classification tasks. mv_classify is a general-purpose function that works on data of arbitrary dimension (e.g., time-frequency data). It performs any combination of cross-validation, searchlight analysis, generalization, and other tasks. Two more specialized functions are provided for convenience: mv_classify_across_time and mv_classify_timextime, assume that the data has a time dimension i.e., it is a 3-D [samples × features × time points] array. mv_classify_across_time performs classification for every time point, resulting in a vector of cross-validated metrics, the length of the vector being the number of time points. mv_classify_timextime expects the same 3-D input. It implements time generalization (King and Dehaene, 2014) i.e., classification for every combination of training and test time points, resulting in a 2-D matrix of cross-validated metrics. For regression tasks, the equivalent to mv_classify is the function mv_regress. It also works with data of arbitrary dimension and supports both searchlight and generalization.

All high-level functions take three input arguments. First, cfg, a configuration structure wherein parameters for the analysis can be set. Second, X, the data acting as input to the model. Third, clabel or y, a vector of class labels or responses. Some of the parameters in the cfg struct are common to all high-level functions:

- cfg.model: name of the classifier or regression model, e.g., 'lda.'
- cfg.hyperparameter: a struct that specifies the hyperparameters for the model. For instance, cfg.hyperparameter.lambda = 0.1 sets the magnitude of shrinkage regularization in LDA. LDA's hyperparameters are introduced in section 2.4.3.
- cfg.metric: specifies the metric to be calculated from the model predictions e.g., classification accuracy or mean-squared error for regression. Metrics are introduced in section 2.6.
- cfg.preprocess: a struct that specifies a nested preprocessing pipeline. The pipeline consists of preprocessing operations that are applied on train and test data separately. Preprocessing is discussed in section 2.3.3.

#### 2.3.1. Cross-Validation

Cross-validation is implemented in all high-level functions. It is controlled by the following parameters that are part of the cfg struct defined in the previous section:

- cfg.cv: cross-validation type, either 'kfold,' 'leaveout,' 'predefined,''holdout,' or 'none'.
- cfg.k: number of folds in k-fold cross-validation.
- cfg.repeat: number of times the cross-validation is repeated with new randomly assigned folds.
- cfg.p: if cfg.cv = 'holdout,'p is the fraction of test samples.
- cfg.fold: if cfg.cv = 'predefined,' fold is a vector of integers that specifies which fold a sample belongs to.
- cfg.stratify: if 1, for classification, the class proportions are approximately preserved in each test fold.

See the function mv_get_crossvalidation_folds for more details.

#### 2.3.2. Hyperparameter Tuning

MVPA-Light tries to automate hyperparameter selection as much as possible. This is done using either reasonable default values, hyperparameter estimators [Ledoit and Wolf (2004) for LDA] or hyperparameter-free regularizers (log-F(1,1) for Logistic Regression). If this is not possible, automated grid search using nested cross-validation can be used for testing out different hyperparameter combinations essentially by brute force. For better performance, bespoke hyperparameter tuning functions are implemented for some classifiers. Otherwise, the generic tuning function mv_tune_hyperparameter is used.

#### 2.3.3. Preprocessing

Preprocessing refers to operations applied to the data prior to training the classifier. To not bias the result, some preprocessing operations (such as Common Spatial Patterns) should be performed in a “nested” fashion. That is, they are performed on the training data first and subsequently applied to the test data using parameters estimated from the training data (Lemm et al., 2011; Varoquaux et al., 2017). Currently implemented functions include PCA, sample averaging (Cichy and Pantazis, 2017), kernel averaging (Treder, 2018), and under-/oversampling for unbalanced data. Preprocessing pipelines are defined by adding the cfg.preprocess parameter. For instance,


cfg.preprocess = {'undersample,' 'zscore,'
 'average_kernel'}


adds a preprocessing pipeline that performs undersampling of the data followed by z-scoring and kernel averaging.

#### 2.3.4. Searchlight Analysis

In MVPA-Light, mv_classify_across_time performs searchlight analysis across the time axis. More bespoke searchlight analyses can be conducted using mv_classify and mv_regress by setting the parameter cfg.neighbours.

### 2.4. Classifiers

**Figure 2:**
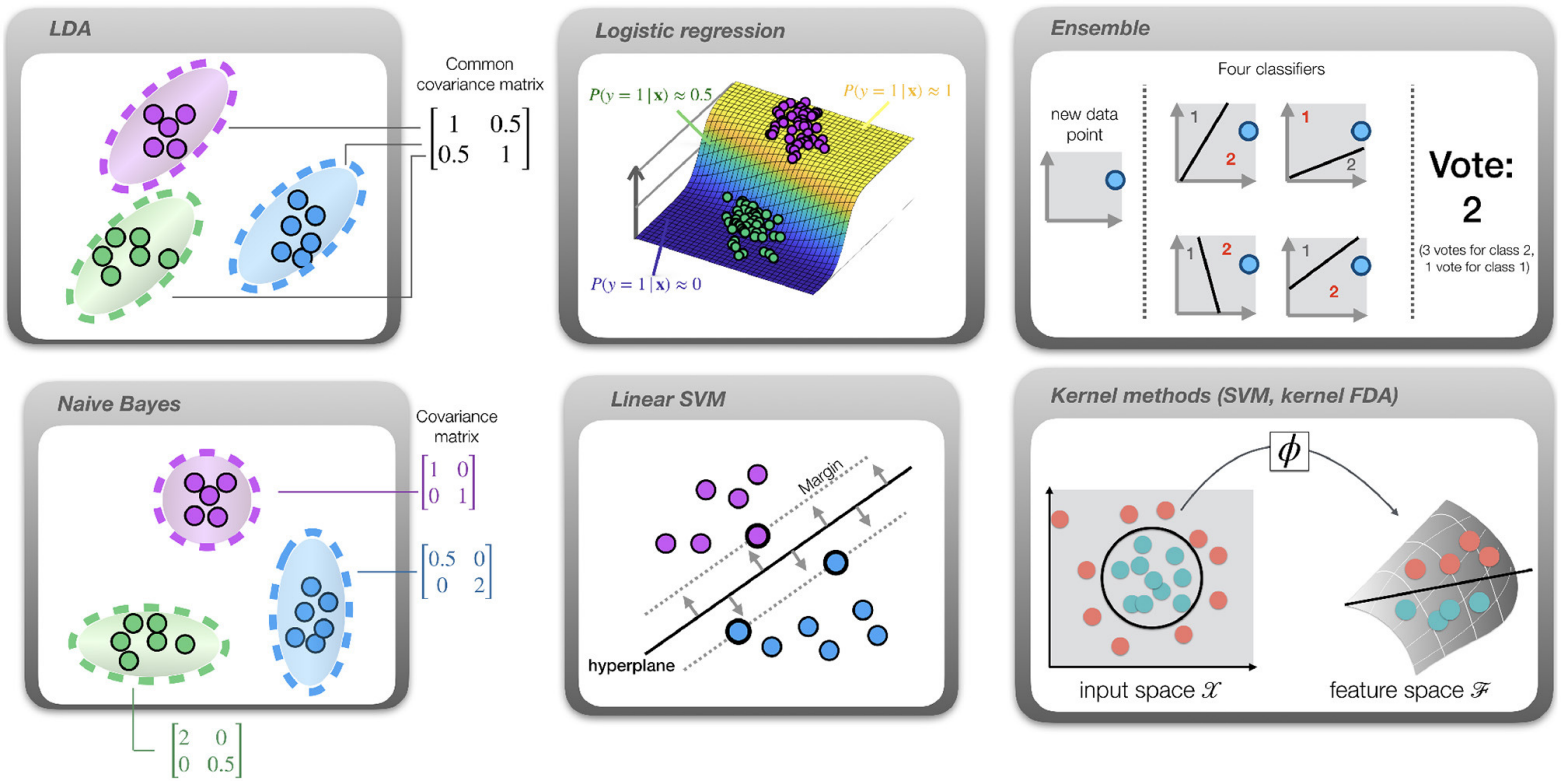
Overview of the available classifiers. Dots represent samples, color indicates the class. *LDA*: different classes are assumed to have the same covariance matrix, indicated by the ellipsoids. *Gaussian Naive Bayes*: features are conditionally independent, yielding diagonal covariance matrices. *Logistic regression*: a sigmoid function (curved plane) is fit to directly model class probabilities. *SVM*: a hyperplane (solid line) is fit such that the margin (distance from hyperplane to closest sample; indicated by dotted lines) is maximized. *Ensemble*: multiple classifiers are trained on subsets of the data. In this example, their hyperplanes partition the data into spaces belonging to classes 1 and 2. After applying all classifiers to a new data point and collecting their “votes,” the class receiving most votes is selected. *Kernel methods*: in this example the optimal decision boundary is circular (circle), hence the data is not linearly separable. After projection into a high-dimensional feature space using a map ϕ, the data becomes linearly separable (solid line) and a linear classifier such as SVM or LDA can be successfully applied in this space.


The main workhorses of MVPA are classifiers and regression models. Figure 2 provides a pictorial description of the classifiers. They are implemented using pairs of train/test functions. In the high-level interface, a classifier and its hyperparameters can be specified using cfg.model and cfg.hyperparameter. For instance,

cfg.model = 'lda';
cfg.hyperparameter.lambda = 0.1;


specifies an LDA classifier and sets the hyperparameter lambda = 0.1. The cfg struct can then be used in a high-level function call, e.g., acc = mv_classify_across_time(cfg, X, clabel). Alternatively, as a low-level interface, the train/test functions can be called directly. For instance, an LDA classifier can be trained directly using


model = train_lda(param, X, clabel)


where X is the training data and clabel are the corresponding class labels. param is a MATLAB struct that contains hyperparameters (same as cfg.hyperparameter). It can be initialized by calling param = mv_get_hyperparameter('lda'). An explanation of the hyperparameters for LDA is given when typing help('train_lda') in MATLAB. The output model is a struct that contains the classifier's parameters after training. The classifier can be applied to test data, denoted as Xtest, by calling


  [clabel, dval, prob] = test_lda(model,
Xtest)


The first output argument clabel is the predicted class labels. They can be compared against the true class labels to calculate a classification performance metric. test_lda provides two additional outputs, but not all classifiers have this capability. dval is the decision value, a dimensionless quantity that measures the distance to the hyperplane. prob contains the probability for a given sample to belong to class 1.

To introduce some mathematical notation needed in the following, data is denoted as a matrix **X** ∈ ℝ^*n* × *p*^ of *n* samples and *p* predictors/features. The i-th row of **X** is denoted as the column vector ${\text{x}}_{i}\in {\mathbb{R}}^{p}$. Class labels are stored in a vector **y** ∈ ℝ^*n*^ with *y*_*i*_ referring to the i-th class label. When the index is not relevant, the feature vector and class label are simply referred to as **x** and *y*. Before describing the classifiers, two conceptual perspectives are introduced that highlight some of their similarities.

#### 2.4.1. Perspective 1: Linear Classifiers

For two classes, linear classifiers such as LDA, Logistic Regression, and linear SVM act on the data in a unified way. The decision value for a test sample **x** is given by

(1) $$\begin{array}{l} \text{dval}={\text{w}}^{⊤}\text{x}+b \end{array}$$

where **w** is the weight vector or normal to the hyperplane specifying the linear combination of features, and *b* is the threshold/bias term. A sample is assigned to the first class if dval> 0 and to the second class if dval < 0. If we encode class 1 as +1 and class 2 as –1, this can be expressed concisely as

$$\begin{array}{l} \text{predicted class}=\text{sign}\left({\text{w}}^{⊤}\text{x}+b\right) \end{array}$$

where sign:ℝ → {−1, +1} is the sign function. Linear classifiers differ only in the way that **w** and *b* are derived.

#### 2.4.2. Perspective 2: Probabilistic Classifiers

Another useful perspective is given by the Bayesian framework (Bishop, 2007). Probabilistic classifiers such as LDA, Naive Bayes, and Logistic Regression are able to directly model class probabilities for individual samples. Let us denote the (posterior) probability for class *i* given test sample **x** as *P*(*y* = *i*|**x**). A possible approach for calculating this quantity is Bayes' theorem:

(2) $$\begin{array}{l} P\left(y=i|\text{x}\right)=\frac{P\left(\text{x}|y=i\right)\;P\left(y=i\right)}{P\left(\text{x}\right)} \end{array}$$

Here, *P*(**x**|*y* = *i*) is the likelihood function which quantifies the relative probability of observing **x** given the class label, and *P*(*y* = *i*) is the prior probability for a sample to belong to class *i*. The denominator, called evidence, can be calculated by marginalizing across the classes: $P\left(\text{x}\right)=\underset{i}{\sum }P\left(\text{x}|y=i\right)\;P\left(y=i\right)$.

#### 2.4.3. Linear Discriminant Analysis (LDA)

If the classes follow a multivariate Gaussian distribution with a common covariance matrix for all classes, LDA yields the theoretically optimal classifier (Duda et al., 2001). In the context of EEG/MEG analysis, LDA is discussed in detail in Blankertz et al. (2011). The likelihood function takes the form

(3) $$\begin{array}{l} P\left(\text{x}|y=i\right)\sim N\left({\text{m}}_{i},\text{Σ}\right) \end{array}$$

i.e., it is multivariate Gaussian distributed with a class-specific mean **m**_*i*_ and common covariance matrix **Σ**. Both need to be estimated from the training data. Equation (2) can then be evaluated to calculate class probabilities. A prediction can be done by selecting the most likely class out of all candidate classes,

$$\begin{array}{l} \text{predicted class}=\underset{i}{\text{arg max}}\;P\left(y=i|\text{x}\right) \end{array}$$

which is known as the maximum a posteriori (MAP) rule. LDA is closely related to other statistical models. For two classes, LDA is equivalent to Linear Regression using the class labels as targets. It is also equivalent to Linearly Constrained Minimum Variance (LCMV) beamforming when applied to ERP data (Treder et al., 2016). The latter equivalence relationship also applies to other methods based on generalized eigenvalue decomposition of covariance matrices (De Cheveigné and Parra, 2014).

In MVPA-Light, multi-class LDA is implemented as the classifier 'multiclass_lda.' For two classes, a more efficient implementation denoted as 'lda' is available. In practice, the covariance matrix is often ill-conditioned and needs to be regularized (Blankertz et al., 2011). The hyperparameter lambda controls the amount of regularization. In shrinkage regularization, lambda ∈ [0, 1] blends between the empirical covariance matrix (lambda = 0) and a scaled identity matrix (lambda= 1). By default, lambda is estimated automatically using the Ledoit-Wolf formula (Ledoit and Wolf, 2004). Section 4.1 (Appendix in Supplementary Material) discusses the implementation of LDA in detail.

#### 2.4.4. Naive Bayes

In Naive Bayes, the features are assumed to be conditionally independent of each other given the class label (Bishop, 2007). While this is indeed naive and often wrong, Naive Bayes has nevertheless been remarkably successful in classification problems. The independence assumption leads to a straightforward formula for the likelihood function since only univariate densities need to be estimated. Let *x*(*j*) be the *j*-th feature and **x** = [*x*(1), *x*(2), …, *x*(*p*)]^⊤^ be a feature vector then the likelihood function is given by

$$\begin{array}{l} P\left(\text{x}|y=i\right)=\overset{p}{\underset{j=1}{\prod }}P\left(x^{\left(j\right)}|y=i\right) \end{array}$$

Like in LDA, the predicted class can be obtained using the MAP rule. In MVPA-Light, Naive Bayes is implemented as 'naive_bayes.' Additionally, MVPA-Light assumes that these densities are univariate Gaussian i.e., $P\left(x\left(j\right)|y=i\right)\sim N\left(m_{ij},\sigma_{ij}^{2}\right)$. For Gaussian densities, the independence assumption is equivalent to assuming that the covariance matrix is diagonal. As indicated in Figure 2, there is a close relationship between LDA and Gaussian Naive Bayes: LDA allows for a dense covariance matrix, but it requires that it is the same for all classes. In contrast, Naive Bayes allows each class to have a different covariance matrix, but it requires each matrix to be diagonal. Additional details on the implementation are given in section 4.2 (Appendix in Supplementary Material).

#### 2.4.5. Logistic Regression

In Logistic Regression for two classes, the posterior probability is modeled directly by fitting a logistic function to the data (Hastie et al., 2009). If the two classes are coded as +1 and –1, it is given by

(4) $$\begin{array}{l} P\left(y=\pm 1|\text{x}\right)=\frac{1}{1+\text{exp}\left(-y\left({\text{w}}^{⊤}\text{x}+b\right)\right)} \end{array}$$

The weights **w** are found by minimizing the logistic loss function

(5) $$\begin{array}{l} L_{\text{LR}}(\text{w})=\sum_{i=1}^{n}\log[1+\text{exp}(-y_{i}({\text{w}}^{⊤}{\text{x}}_{i}+b))] \end{array}$$

In MVPA-Light, Logistic Regression is implemented as 'logreg.' By default, log-F(1,1) regularization (reg = 'logf') is used by imposing Jeffrey's prior on the weights (Firth, 1993; King and Zeng, 2001; Rahman and Sultana, 2017). Alternatively, L2-regularization can be used to impose a Gaussian prior on the weights (reg = 'l2'). In this case, an additional hyperparameter lambda ∈ [0, ∞) that controls the amount of regularization needs to be specified by the user. It can be set to a fixed value. Alternatively, a range of candidates can be specified (e.g., lambda = [0.001, 0.01, 0.1, 1]). A nested cross-validation is then performed to select the optimal value. Additional details on the implementation are given in section 4.3 (Appendix in Supplementary Material). An alternative implementation using LIBLINEAR is also available, see Section 2.9.

#### 2.4.6. Linear Support Vector Machine (SVM)

A SVM has no underlying probabilistic model. Instead, it is based on the idea of maximizing the margin (Hearst et al., 1998; Schölkopf and Smola, 2001). For linearly separable data, the margin is the distance from the hyperplane to the closest data point (dotted line in Figure 2). This distance is given by 1/||**w**||. Minimizing ||**w**|| is then equal to maximizing the margin. At the same time, one needs to make sure that the training samples are correctly classified at a distance from the hyperplane. This is achieved by requiring ${\text{w}}^{⊤}{\text{x}}_{i}+b\geq 1$ for class 1 and ${\text{w}}^{⊤}{\text{x}}_{i}+b\leq -1$ for class 2. Encoding the classes as +1 and –1, both terms can be combined into $y_{i}\left({\text{w}}^{⊤}{\text{x}}_{i}+b\right)\geq 1$. This constraint cannot be satisfied for every training sample *i* ∈ {1, …, *n*} if the data cannot be perfectly separated. Therefore, positive slack variables ξ_*i*_ are introduced that allow for misclassifications. Now the goal becomes to maximize the margin while simultaneously minimizing the amount of constraint violations given by $\underset{i}{\sum }\xi_{i}$. Put together, this leads to the following optimization problem:

(6) $$\begin{array}{l} \begin{array}{c} \underset{\text{w}}{\text{arg min}}\;\frac{1}{2}\|\text{w}{\|}^{2}+c\underset{i}{\sum }\xi_{i}\\ \text{subject to }\forall i:y_{i}\left({\text{w}}^{⊤}{\text{x}}_{i}+b\right)\geq 1-\xi_{i}\\ \;\forall i:\xi_{i}\geq 0 \end{array} \end{array}$$

The resultant classifier, called two-class L1-Support Vector Machine (SVM) is implemented as ‘svm.' The hyperparameter c controls the amount of regularization and needs to be set by the user. Despite the lack of a probabilistic model, a Platt approximation using an external function (http://www.work.caltech.edu/htlin/program/libsvm/) is used to estimate class probabilities if required. Additional details on the implementation are given in section 4.4 (Appendix in Supplementary Material). Alternative implementations using LIBSVM and LIBLINEAR are also available, see section 2.9.

#### 2.4.7. Kernel Classifiers

In kernel methods such as SVM and kernel FDA, a sample is implicitly mapped from the input space X into a high-dimensional feature space F using a map ϕ:X→F. As illustrated in Figure 2, such a map can translate a non-linear classification problem into a linear problem in feature space (Schölkopf and Smola, 2001). For two classes, decision values are given by

(7) $$\begin{array}{l} \text{dval}={\text{w}}_{\phi }^{⊤}\phi \left(\text{x}\right)+b \end{array}$$

where **w**_ϕ_ is the weight vector in feature space. If we compare this formula to Equation (1), it becomes evident that kernel classifiers are linear classifiers acting on non-linear transformations of the features. Often, it is infeasible to explicitly apply the map due to the high dimensionality of F. However, for methods such as SVM and LDA, an efficient workaround is available. The optimization problem can be rewritten into a form wherein only the inner products between pairs of samples are needed, i.e., 〈ϕ(**x**), ϕ(**x**′)〉 for samples **x** and **x**′. Now, if ϕ maps to a Reproducing Kernel Hilbert Space (RKHS), these inner products can be efficiently calculated via a kernel function *k* that operates in input space, resulting in the identity *k*(**x**, **x**′) = 〈ϕ(**x**), ϕ(**x**′)〉. This is known as the *kernel trick*.

To give a simple example, consider two samples with two-dimensional features, **x** = [*x*_1_, *x*_2_] and ${\text{x}}^{\prime }=\left[x_{1}^{\prime },x_{2}^{\prime }\right]$. The homogeneous polynomial kernel of degree 2 has the kernel function $k\left(\text{x},{\text{x}}^{\prime }\right)={\left(\overset{2}{\underset{i=1}{\sum }}x_{i}x_{i}^{\prime }\right)}^{2}$ and the corresponding feature map ϕ:ℝ^2^ → ℝ^3^ with $\phi \left(\text{x}\right)=\left[x_{1}^{2},\sqrt{2}x_{1}x_{2},x_{2}^{2}\right]$. It is now easily verified that *k*(**x**, **x**′) = 〈ϕ(**x**), ϕ(**x**′)〉. For LDA, a kernelized version called Kernel Fisher Discriminant Analysis (KFDA) has been developed by Mika et al. (1999). It is available as 'kernel_fda.' By default, the model is regularized using shrinkage regularization controlled by the hyperparameter lambda. Often, a small value (e.g., lambda = 0.01) is adequate. Additional details on the implementation are given in section 4.5 (Appendix in Supplementary Material). For kernel SVM, either 'svm' or the LIBSVM interface can be used. For both SVM and KFDA, the kernel can be chosen by setting the kernel parameter. Further information on the kernels is provided in the train functions.

#### 2.4.8. Ensemble Methods

An 'ensemble' is a meta-classifier that trains dozens or even hundreds of classifiers. In ensembles, these individual classifiers are referred to as learners. The type of learner can be set using the learner hyperparameter. For instance, setting learner = 'svm' creates an ensemble of SVM classifiers. To encourage the learners to focus on different aspects of the data, every learner is presented just a subset of the training data. nsamples controls the number of training samples that is randomly selected for a given learner, whereas nfeatures controls the number of features. The final classifier output is determined via a voting strategy. If strategy = 'vote,' then the class label produced by each individual learner serves as a vote. The class that receives the maximum number of votes is then selected. If strategy = 'dval' then the raw decision values are averaged and the final decision is taken based on whether the average is positive or negative. The latter only works with classifiers that produce decision values.

#### 2.4.9. Classifier Output Type

For every test sample, a classifier produces raw output. This output takes either a discrete form as a *class label* or a continuous one. If it is continuous, it comes either as a *decision value* or as a *probability*. A decision value is an unbounded number that can be positive or negative. Its absolute value corresponds to the distance to the hyperplane. For two classes, the probability is a number between 0 and 1 representing the probability that a sample belongs to class 1. In the high-level interface, the classifier output can be specified explicitly by setting cfg.output_type to 'clabel,''dval,' or 'prob.' In most cases, however, it suffices to let MVPA-Light infer the output type.

### 2.5. Regression Models

Like classifiers, regression models are implemented using pairs of train/test functions. In the high-level function mv_regress, a regression model is specified using the cfg.model parameter. Low-level access is possible by directly calling the train/test functions. For instance, model = train_ridge(param, X, y) trains a ridge regression model. X is the training data and y are the corresponding responses. param is a MATLAB struct that contains hyperparameters. The output model is a struct that contains the model parameters after training. The model can be applied to test data by calling yhat = test_ridge(model, Xtest) where Xtest is test data. The output of the test function is the model predictions. In the following section, the individual regression models are introduced. It is assumed that the training data is contained in matrix **X** ∈ ℝ^*n* × *p*^ of *n* samples and *p* predictors. The i-th row of this matrix is denoted as the column vector ${\text{x}}_{i}\in {\mathbb{R}}^{p}$. Responses are stored in a vector **y** ∈ ℝ^*n*^ with **y**_*i*_ referring to the i-th response.

#### 2.5.1. Perspective: Linear Regression

Linear models such as Linear Regression, Ridge Regression, and linear Support Vector Regression, act on the data in a unified way by means of a vector of coefficients **w** (often represented by β's in the literature). Linear regression models differ only in the way that **w** is derived. To simplify the notation, it is assumed that the data matrix **X** contains a column of ones and hence the intercept term is contained in **w**. For a test sample **x**, the predicted response is given by $\hat{y}={\text{w}}^{⊤}\text{x}$. The vector of predicted responses on the training data $\hat{\text{y}}\in {\mathbb{R}}^{n}$ can be written in matrix notation as

(8) $$\begin{array}{l} \hat{\text{y}}=\text{X}\text{w} \end{array}$$

During training, the goal is to find a **w** such that $y_{i}\approx {\hat{y}}_{i}$ for each training sample. A natural measure of closeness between the true response and the prediction is the squared distance ${\left(y_{i}-{\hat{y}}_{i}\right)}^{2}$, which directly leads to the sum of squares measure $\overset{n}{\underset{i=1}{\sum }}{\left(y_{i}-{\hat{y}}_{i}\right)}^{2}$. In matrix notation, the sum of squares is denoted as

(9) $$\begin{array}{l} L_{\text{OLS}}\left(\text{w}\right)=\|\text{y}-\text{X}\text{w}{\|}^{2} \end{array}$$

The solution that minimizes this quantity, known as ordinary least squares (OLS) solution to linear regression, is given by **w** = (**X**^⊤^**X**)^−1^ **X**^⊤^**y**. It is worth noting that if one divides the sum of squares by the number of samples *n*, one obtains the regression metric *mean squared error* (MSE).

#### 2.5.2. Ridge Regression

Ridge regression is a regularized version of OLS regression. It is useful for data that suffers from multicollinearity. The model is regularized by adding a L2 penalty that shrinks the weights toward zero. For a given regularization parameter lambda ∈ [0, ∞), denoted by the Greek symbol λ, the loss function is given by

(10) $$\begin{array}{l} L_{\text{ridge}}\left(\text{w}\right)=\|\text{y}-\text{X}\text{w}{\|}^{2}+\lambda \|\text{w}{\|}^{2} \end{array}$$

This convex optimization problem can be solved directly by calculating the gradient and setting it to zero. Alternatively, it can be rewritten into its dual Lagrangian form first (Bishop, 2007). The resultant primal and dual ridge solutions that minimize the loss function are given by

(11) $$\begin{array}{l} \begin{array}{c} \text{w}=\;{\left({\text{X}}^{⊤}\text{X}+\lambda I_{p}\right)}^{-1}\;{\text{X}}^{⊤}\text{y}\;(\text{primal solution})\\ =\;{\text{X}}^{⊤}{\left(\text{X}{\text{X}}^{⊤}+\lambda I_{n}\right)}^{-1}\;\text{y}\;\text{(dual solution)} \end{array} \end{array}$$

where $I_{p}\in {\mathbb{R}}^{p\times p}$ and $I_{n}\in {\mathbb{R}}^{n\times n}$ are identity matrices. The equivalence between the primal and dual solution can be verified by left-multiplying both solutions with $\left({\text{X}}^{⊤}\text{X}+\lambda I_{p}\right)$.

For lambda= 0 ridge regression reduces to OLS regression. By default (form = 'auto'), MVPA-Light dynamically switches between the primal and the dual form depending on whether *n* is larger or smaller than *p*.

#### 2.5.3. Kernel Ridge Regression

Analogous to kernel classifiers (section 2.4.7), a non-linear version of ridge regression can be developed by applying a non-linear transformation to the features. Let this transformation be represented by ϕ:X→F, a map from input space to a Reproducing Kernel Hilbert Space, and $\Phi \left(\text{X}\right)={\left[\phi \left({\text{x}}_{1}\right),\phi \left({\text{x}}_{2}\right),\ldots ,\phi \left({\text{x}}_{n}\right)\right]}^{⊤}$. The solution is given by replacing **X** by Φ(**X**) in Equation (11),

(12) $$\begin{array}{l} \begin{array}{c} {\text{w}}_{\phi }={\left(\Phi {\left(\text{X}\right)}^{⊤}\Phi \left(\text{X}\right)+\lambda \text{I}\right)}^{-1}\;\Phi {\left(\text{X}\right)}^{⊤}\text{y}\;\text{(primal solution)}\\ \;=\Phi {\left(\text{X}\right)}^{⊤}{\left(\Phi \left(\text{X}\right)\Phi {\left(\text{X}\right)}^{⊤}+\lambda {\text{I}}_{n}\right)}^{-1}\;\text{y}\;\text{(dual solution)} \end{array} \end{array}$$

Unfortunately, this solution is of limited practical use, since generally speaking the feature space is too high-dimensional to represent **w**_ϕ_ and Φ(**X**). However, the dual solution can be rewritten as follows. Let **K** = Φ(**X**)Φ(**X**)^⊤^ be the kernel matrix with **K**_*ij*_ = *k*(**x**_*i*_, **x**_*j*_) for a kernel function *k*. Define the vector of dual weights ***α*** as

(13) $$\begin{array}{l} \begin{array}{c} \alpha ={\left(\text{K}+\lambda I_{n}\right)}^{-1}\;\text{y}. \end{array} \end{array}$$

Then the predicted response to a test sample **x** can be rewritten in terms of kernel evaluations:

(14) $$\begin{array}{l} \begin{array}{c} f\left(\text{x}\right)={\text{w}}_{\phi }^{⊤}\phi \left(\text{x}\right)=\alpha^{⊤}\Phi \left(\text{X}\right)\phi \left(\text{x}\right)=\overset{n}{\underset{i=1}{\sum }}\alpha_{i}k\left({\text{x}}_{i},\text{x}\right). \end{array} \end{array}$$

### 2.6. Performance Metrics

In most cases, the quantity of interest is not the raw model output but rather a metric that summarizes the performance of the classifier or regression model on test data. The desired metric can be specified by e.g., setting cfg.metric = 'accuracy' in any high-level function. Multiple metrics can be requested by providing a cell array, e.g., cfg.metric = {'accuracy,' 'auc'}. Table 1 lists the metrics implemented in MVPA-Light. For a thorough discussion of classification metrics, refer to Sokolova and Lapalme (2009).

**Table 1:** Metrics in MVPA-Light.

**Task**	**Metric**	**Range**	**Description**
Classification	'accuracy'	[0,1]	Fraction correctly predicted class labels.
	'auc'	[0,1]	For two classes only. An alternative to classification accuracy that is more robust to imbalanced classes. Requires continuous classifier output (decision values or probabilities). 0.5 means chance-level performance and 1 means perfect separation of the classes.
	'confusion'	[0,1]	Confusion matrix. Rows corresponds to true class, columns to predicted class. The (*i, j*)-th element gives the proportion of samples of class i that have been classified as class j.
	'dval'	(−∞, +∞)	For two classes only. Average decision value, for each class separately.
	'f1'	[0,1]	Combines precision (PR) and recall (R) into a single score using the harmonic average 2*PR*R / (PR+R).
	'kappa'	[-1, 1]	Cohen's kappa, a measure of inter-rater reliability.
	'precision'	[0,1]	TP / (TP + FP). Fraction of samples labeled as positive that actually belong to the positive class. For multi-class, it is calculated per class from the confusion matrix.
	'recall'	[0,1]	TP / (TP + FN). Fraction of positive samples that have been detected. For multi-class, it is calculated per class from the confusion matrix.
	'tval'	(−∞, +∞)	For two classes only. T-test statistic for the unequal sample size, equal variance case, based on decision values.
	'none'	(−∞, +∞)	Returns a cell array with the raw classifier outputs for all test sets.
Regression	'mae'	[0, ∞)	Mean absolute error: $1/n\overset{n}{\underset{i=1}{\sum }}|y_{i}-{ŷ}_{i}|$.
	'mse'	[0, ∞)	Mean squared error: $1/n\overset{n}{\underset{i=1}{\sum }}{\left(y_{i}-{ŷ}_{i}\right)}^{2}$.
	'r_squared'	(−∞, 1]	*R*^2^ coefficient representing the fraction of variance explained by the model.

*TP, true positives; FP, false positives; FN, false negatives. Regression: y = responses, ŷ = model predictions*.

If cross-validation is used then the metric is initially calculated for each test set in each repetition separately. It is then averaged across test sets and repetitions. Since the number of samples in a test set can vary across different folds, a proportionally weighted average is used whereby larger test sets get a larger weight.

### 2.7. Statistical Analysis

In neuroimaging experiments, establishing the statistical significance of a metric is often more important than maximizing the metric *per se*. Neuroimaging data is typically hierarchical: a study comprises many subjects, and each subject comprises many trials. To perform group analysis, a common approach is then to start with a level 1 (single-subject) analysis and calculate a classification or regression metric. At this stage, the samples consist of single trials for a particular subject. The metrics are then taken on to level 2 (group level). At this stage, each subject constitutes one sample (Mumford and Poldrack, 2007). The function mv_statistics implements both level 1 (single-subject) and level 2 (group level) statistical analysis. For level 1 analysis, the following tests are available:

- Binomial test: uses a binomial distribution to calculate the p-value under the null hypothesis that classification accuracy is at chance. Requires classification accuracy as metric.
- Permutation test: non-parametric significance test. Creates a null distribution by shuffling the class labels or responses and repeating the multivariate analysis e.g., 1,000 times.
- Cluster permutation test: an elegant solution to the multiple comparisons problems arising when MVPA is performed along multiple dimensions (e.g., for each time-frequency point). Uses the cluster statistic introduced in Maris and Oostenveld (2007).

For level 2 analysis, a permutation test (with and without cluster correction) is available for within-subject and between-subjects designs. Note that no classification/regression is performed. The metrics that have been obtained in the level 1 analysis for each subject are simply subjected to a standard statistical test. In the *within-subject* design, two different cases are considered. If pairs of values have been observed (e.g., mean decision values for class 1 and 2) they are tested for a significant difference across subjects. If only one value has been observed (e.g., AUC) it is tested against a given null value (e.g., 0.5). As test statistics, mean, *t*-test, or Wilcoxon signed-rank test can be used. To create a null distribution, data is permuted by randomly swapping the pairs of values or swapping the value and its null value. In *between-subjects* design, subjects are partitioned into two different groups. The test statistic quantifies whether the metric differs between two groups. A null distribution is created by randomly assigning subjects to groups.

To illustrate this with an example, consider the “faces vs. houses” experiment. For the within-subject design, assume the mean decision values for houses and faces have been determined for each subject using cross-validation. A paired-samples *t*-test across subjects comparing the decision value for faces vs houses is used to calculate a *t*-statistic. A null distribution is created by randomly swapping face and house values for each subject and recomputing the statistic. For a between-subjects design, assume the experiment has also been carried out with a clinical group of Parkinson's patients and AUC values have been recorded for both groups. A Wilcoxon rank sum test is used to compare the AUC for the two groups at each voxel. A null distribution is created by randomly assigning subjects to either the clinical or the control group.

### 2.8. Custom Classifiers and Regression Models

MVPA-Light can be extended with custom models. To this end, the appropriate train and test functions need to be implemented. Additionally, default hyperparameters need to be added to the function mv_get_hyperparameter. In the appendix, it is shown how to implement a prototype classifier that assigns a sample to the closest class centroid.

### 2.9. LIBSVM and LIBLINEAR

LIBSVM (Chang et al., 2011) and LIBLINEAR (Fan et al., 2008) are two high-performance libraries for SVM, Support Vector Regression (SVR), and Logistic Regression. In order to use the libraries with MVPA-Light, the user needs to follow the installation instructions on the respective websites. In particular, the C-code needs to be compiled and added to the MATLAB path. In MVPA-Light, the models are denoted as 'libsvm' and 'liblinear.'

### 2.10. FieldTrip Integration

The FieldTrip (Oostenveld et al., 2011) function ft_statistics_mvpa provides a direct interface between FieldTrip and MVPA-Light. In brief, the function calls MVPA-Light functions to carry out multivariate analysis, and then stores the results back into FieldTrip structs. To use MVPA-Light from high-level FieldTrip functions such as ft_timelockstatistics, one has to set the parameter cfg.method = 'mvpa.' The interface is introduced in detail in a tutorial on the FieldTrip website ^1^.

### 2.11. Development

To maintain the integrity of the toolbox, the unittests/ subfolder features a unit testing framework for all models, optimization algorithms, high-level functions and some of the important utility functions. The unit tests make use of both the example EEG data, random noise, and simulated data. Unit testing can be triggered by executing the run_all_unittests function.

### 2.12. Analysis of a MEEG Dataset

To illustrate MVPA-Light on a real dataset, a multivariate analysis was conducted on a multi-subject, multi-modal face processing dataset wherein subjects viewed images of famous faces, familiar faces, or scrambled faces. See Wakeman and Henson (2014, 2015) for a detailed description of the data. The dataset contains 16 subjects with EEG and MEG simultaneously recorded. The MEEG data was preprocessed using FieldTrip. It was low-pass filtered with a cut-off of 100 Hz and high-pass filtered using a FIR one-pass zero-phase filter with a cut-off of 0.1 Hz. A bandstop filter was applied at 50 Hz to suppress line noise. Subsequently, data was downsampled to 220 Hz and for each subject, the 6 separate runs were combined into a single dataset, yielding 880–889 trials per subject with roughly equal proportions for the three classes. All trials displaying famous faces were coded as class 1, familiar faces as class 2, and scrambled faces as class 3. MVPA was performed to investigate the following questions:

1. ERP classification: Wakeman and Henson (2015) found two prominent event-related components, a N170 and a sustained component roughly starting at 400 ms post-stimulus. Cross-validation with a multi-class classifier was used to investigate whether these components discriminate between the three classes.
2. Time classification: Is there more discriminative information in MEG than in EEG? To answer this, classification across time was performed for three different channel sets, namely EEG only, MEG only, and EEG+MEG combined.
3. Time-frequency classification: Is the discriminative information for famous vs scrambled faces confined to specific oscillatory frequencies and times? To answer this, time-frequency spectra were calculated for single trials and classification was performed at each time-frequency bin separately.
4. Generalization: Are representations shared across time (King and Dehaene, 2014) or frequency? To answer this, time generalization (time x time classification) was applied to the ERF data, and frequency generalization (frequency x frequency classification) was applied to the time-frequency data.

MVPA was performed at the sensor level using a LDA classifier. All analyses were cross-validated using 5- or 10-fold cross-validation. Only the MEG channels were used as features except for analysis 2, where different sets of channels were compared. To assess statistical significance, the following tests were carried out:

- Level 1 statistics. For each subject, the statistical significance of the time generalization (famous vs. scrambled faces) was investigated. For illustrative purposes, the three statistical tests contained in MVPA-Light were compared: binomial, permutation, and cluster permutation tests. Permutation tests were based on 500 random permutations of the class labels. The cluster permutation test was corrected for multiple comparisons by using a cluster statistic, the other tests were uncorrected. For the cluster statistic, a critical value of 0.6 was chosen for classification accuracy. This analysis is reported only for the first subject.
- Level 2 statistics (across subjects). The AUC values obtained in the time-frequency classification analyses were statistically compared to a null value of 0.5 using cluster permutation tests based on a within-subject design.

### 2.13. Analysis of a fMRI Dataset

To illustrate the application of MVPA-Light to fMRI data, another analysis was conducted using a block-design fMRI study. See Haxby et al. (2001) for a detailed description. The dataset was downloaded from http://www.pymvpa.org/datadb/haxby2001.html. The study investigates face and object representations in human ventral temporal cortex. It comprises 6 subjects with 12 runs per subject. In each run, subjects viewed grayscale images of 8 living and non-living object categories, grouped in 24 s blocks separated by rest periods. Images were shown for 500 ms followed by a 1,500 ms inter-stimulus interval. Full-brain fMRI data were recorded with a volume repetition time of 2.5 s. Hence, a stimulus block was covered by roughly 9 volumes. A zero-phase Butterworth high-pass filter with a cut-off frequency of 0.01 Hz was applied in order to remove slow drifts. No other preprocessing was performed. The following questions were addressed:

1. Confusion matrix: Which image categories lead to similar brain activation patterns?
2. Time classification: How does classification performance evolve across time following stimulus onset?
3. Searchlight analysis: Which of the brain regions contain discriminative information that discerns between faces and houses?

Leave-one-run-out cross-validation was used to calculate classification performance. Multi-class LDA with 8 classes served as a classifier. For the searchlight analysis, binary LDA contrasting faces vs. houses was used with AUC serving as metric. The searchlight consisted of a 3x3x3 cube of voxels that was centered on each target voxel. A level 2 cluster permutation test was computed on the AUC values against the null hypothesis that AUC equals 0.5.

### 2.14. Benchmarking

Multivariate analyses can involve hundreds or even thousands of train/test iterations. Therefore, training time (the amount of time required to train a single model on data) is a relevant quantity when evaluating different model implementations. To benchmark MVPA-Light's models, their training time was compared to models in the MATLAB Statistics Toolbox as well as models in Python (Scikit Learn package) and R (different packages). The comparison to other MVPA toolboxes is of less relevance since they often rely on external packages such as LIBSVM and LIBLINEAR which are also available in MVPA-Light (this applies to e.g., DDTBOX, PRoNTo, TDT). The following three datasets were considered:

- MEG single-subjects. The Wakeman and Henson (2015) dataset was used with the famous vs. scrambled faces conditions, epoched in the range [–0.2, 1] s. Data dimensions were 585–592 trials per subject, 306 channels, and 265 time points. MVPA was performed for every subject and every time point separately, using channels as features.
- MEG super-subject. Trials of all subjects in the MEG single-subjects data were concatenated to form a single “super-subject” comprising 9,421 trials, 306 channels, and 265 time points. MVPA was performed for every time point separately, using channels as features.
- fMRI. For each subject in the Haxby et al. (2001) data, all voxels with a non-zero signal were concatenated to a single feature vector. The time dimension was dropped, different time points within a trial were simply considered as different samples. The two classes “face” and “house” were considered, yielding a data matrix of 216 samples (198 samples for subject 5) and between 163,665 and 163,839 voxels per subject. MVPA was performed for every subject separately, using voxels as features.

The MEG single-subjects dataset is of standard size for neuroimaging data and thus serves as a benchmark for ordinary operation. The other two datasets are intended to test the computational limits of the models by using either a large number of trials (MEG super-subject) or a large number of features (fMRI). For the single-subjects dataset, classification performance was measured in addition to training time. To be as unbiased as possible, hyperparameters were mostly unchanged except when a change made the models more comparable across toolboxes (e.g., setting the same regularization value). No hyperparameter tuning was performed in order to quantify pure training time.

The MVPA-Light models were compared to LIBSVM, LIBLINEAR, and MATLAB 2019a's fitcdiscr (LDA), lassoglm (LogReg), fitcnb (Naive Bayes), fitcsvm (SVM), ridge, and fitrsvm (SVR). Python and R-based toolboxes were installed in virtual environments using Anaconda 4.7.12. Scikit Learn 0.21.2 was used together with Python 3.7.3. R version 3.6.1 was used with packages MASS (LDA), glmnet (LogReg and Ridge), e1071 (Naive Bayes, SVM, SVR), and listdtr (Kernel Ridge).

For the single-subject data, the timing results were averaged across subjects. Then for both the single-subject and the super-subject, mean and standard deviation was calculated across time points. For the fMRI data, mean and standard deviation was calculated across subjects. All analyses were conducted after a fresh restart of a desktop computer with networking disabled. The computer had an Intel Core i7-6700 @ 3.40 GHz x 8 CPU with 64 GB RAM running on Ubuntu 18.04. All scripts are available in the accompanying GitHub repository^2^.

### 2.15. Results

#### 2.15.1. MEEG Data

**Figure 3:**
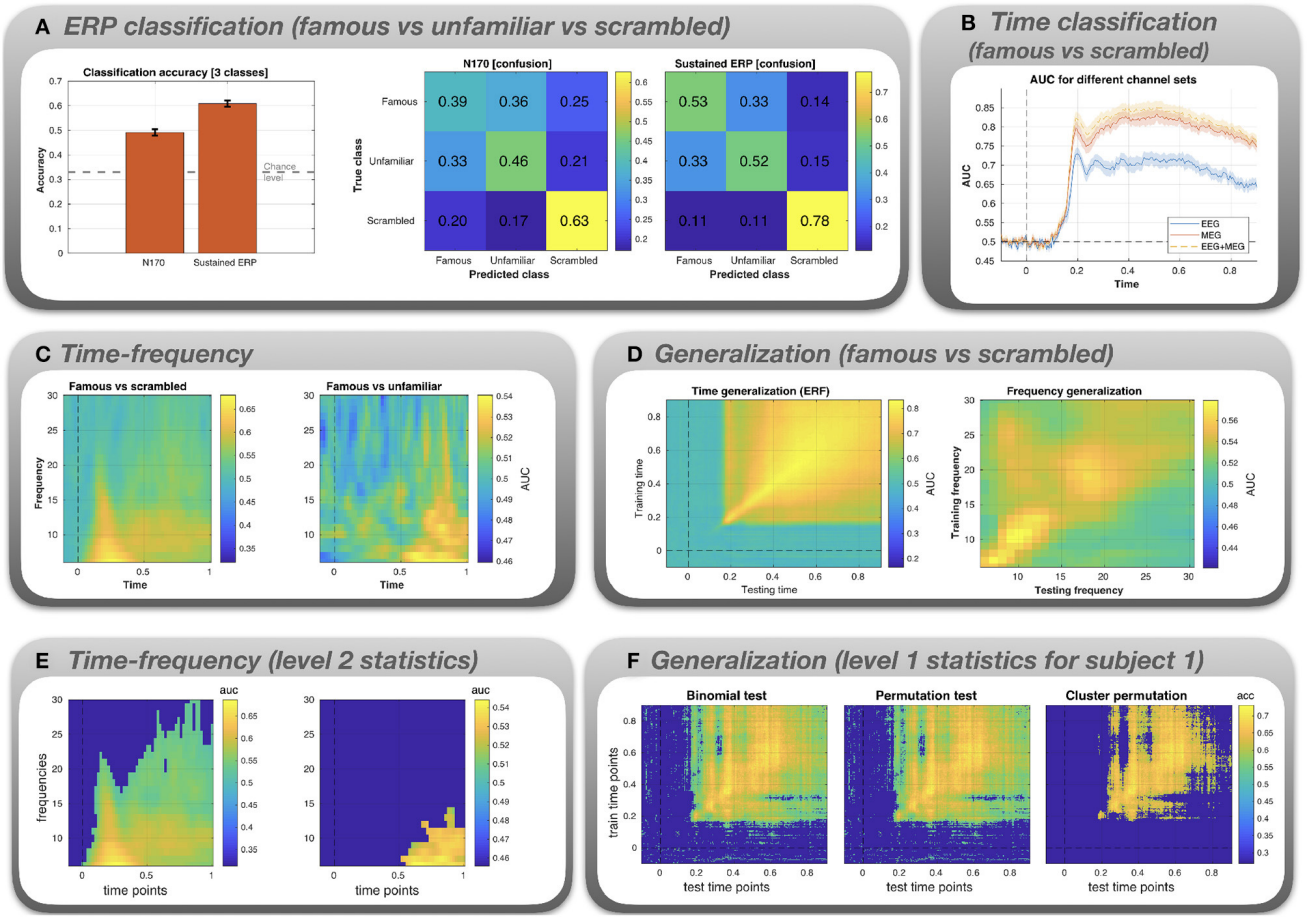
Results for the classification analysis of the Wakeman and Henson (2015) MEEG data. **(A)** Multi-class classification (famous vs. unfamiliar vs. scrambled faces) of N170 and sustained ERP component. **(B)** AUC is plotted as a function of time for famous vs. scrambled images. The classification was performed using three different channel sets: EEG only, MEG only, and EEG+MEG combined. **(C)** Binary classification (famous vs. scrambled and famous vs unfamiliar) for time-frequency data. AUC is plotted as a function of both time and frequency. The AUC values are color-coded. **(D)** Time x time generalization and frequency x frequency generalization using a binary classifier (famous vs. scrambled). **(E)** Level 2 statistical analysis of the time-frequency classification. **(F)** Level 1 statistical analysis of the time x time generalization, shown exemplarily for subject 1.

Figure 3 depicts the results of the MVPA, averaged across subjects. Errorbars depict standard error across subjects.

*ERP classification* (Figure 3A). The bar graph shows that for both the N170 and the sustained ERP component classification accuracy is significantly above the chance level of 33%. Accuracy can be broken down into confusion matrices that show which combinations of classes get misclassified (“confused”). For both N170 and the sustained ERP component, the highest accuracy is obtained for the scrambled images (0.63 and 0.78). Moreover, misclassification (off-diagonal elements) is most prominent for the famous and unfamiliar faces. This is not surprising since both types of images are identical in terms of low-level features and both show actual faces, in contrast to the scrambled images.

*Time classification* (Figure 3B). The classes are not discriminable prior to the occurrence of the N170. A classification peak at the time of the N170 can be seen for all channel sets. At this stage, the AUC values diverge, with EEG yielding a significantly lower AUC. Combining EEG+MEG seems to yield a slightly higher performance than MEG alone.

*Time-frequency classification* (Figure 3C). For famous vs scrambled faces, peak performance is reached in the delta frequency band at a latency between 0.2 and 0.4 s. For famous vs unfamiliar faces, peak performance is attained in the latter half of the trial (0.5–1 s) in the theta and alpha frequency bands.

*Generalization* (Figure 3D). The first plot depicts AUC (color-coded) as a function of training time (y-axis) and testing time (x-axis). There is evidence for widespread time generalization for famous vs scrambled faces starting about at the time of the N170 peak and covering most of the remaining trial. In particular, there is generalization between the N170 and the later sustained component (horizontal and vertical lines emanating at 0.17 s), suggesting some correlation between the spatial pattern of the N170 and the sustained component. The second plot depicts AUC as a function of frequency. There is some generalization in the theta band (lower-left corner), the alpha band, and the lower beta band (16–22 Hz). Also, when the classifier is trained in the beta band, classification performance partially generalizes to the alpha band. However, the overall performance is low when compared to the time-locked data.

*Level 2 statistics* (Figure 3E). Group statistical analysis based on the time-frequency classification data in the panel above. Images depict AUC values masked by significance (deep blue = not significant). For the famous vs. scrambled faces classification, a large cluster spanning the whole trial and especially the low frequency bands is evident. For the famous vs. unfamiliar faces condition, there is a significant cluster corresponding to large AUC values evident after 0.5 s and confined to the lower frequency range.

*Level 1 statistics* (Figure 3F). Level 1 statistical analysis based on the time generalization data in the panel above, shown exemplarily for subject 1. Images depict the AUC values masked by significance. Both uncorrected tests (binomial and permutation test) exhibit spurious effects even at pre-stimulus time. Most of these spurious effects disappear under the cluster permutation test.

#### 2.15.2. fMRI Data

**Figure 4:**
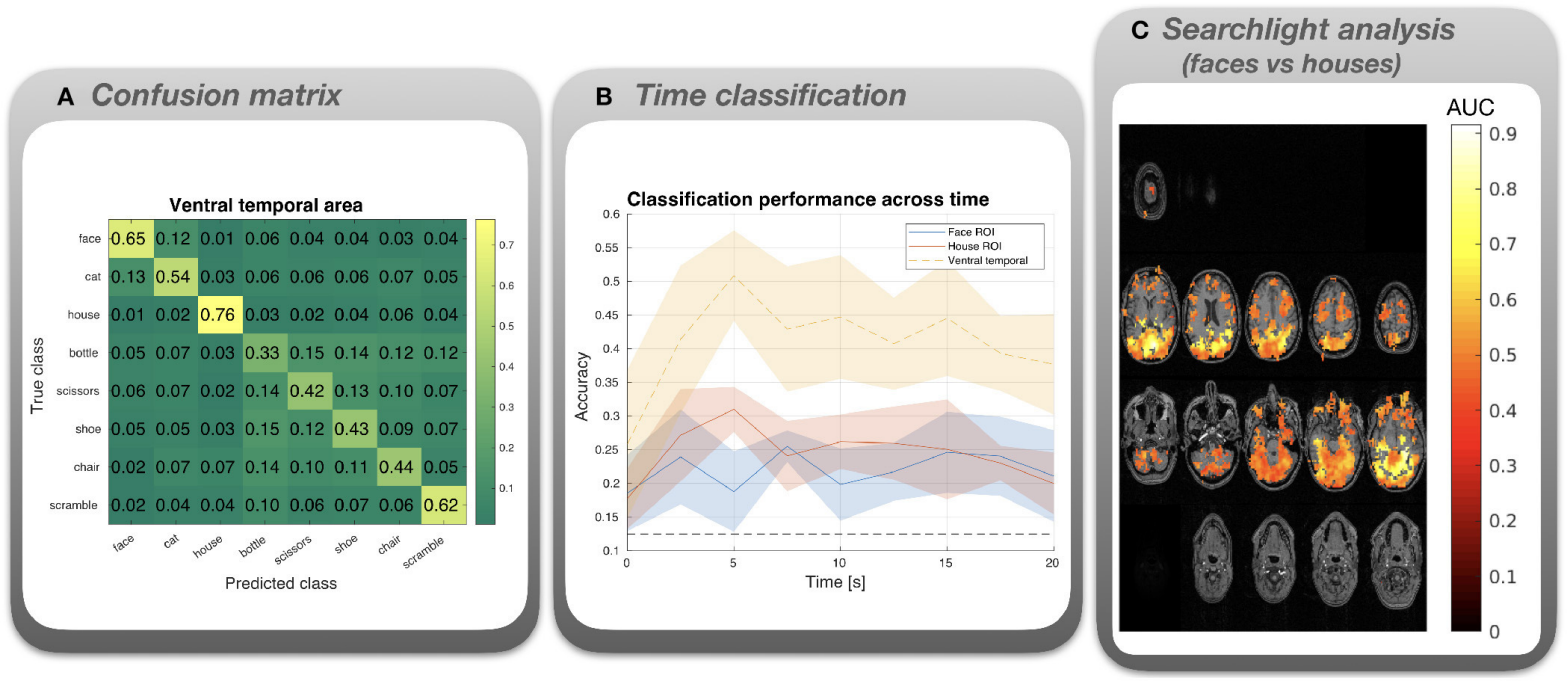
Results for the classification analysis of the Haxby et al. (2001) fMRI data. **(A)** Confusion matrix for multi-class (8 classes) classification based on voxels in the ventral temporal area, averaged across subjects. **(B)** Multi-class (8 classes) classification accuracy was calculated for each time point following stimulus onset. Lines depict means across subjects, shaded areas correspond to standard error. Masks were used to select voxels in the ventral temporal area (yellow line), voxels responsive to faces (blue), or voxels responsive to houses (red). **(C)** Cluster permutation test results based on a searchlight analysis using a binary classifier (faces vs houses). Red spots represent AUC values superimposed on axial slices of the averaged structural MRI. All depicted AUC values correspond to the significant cluster; other AUC values have been masked out.

Figure 4 depicts the results of the MVPA on the fMRI data, averaged across subjects.

*Confusion matrix* (Figure 4A). A mask provided with the data was applied to select voxels from ventral temporal areas. A high overall performance is observed for LDA with 8 classes. Misclassifications tend to be confined to general semantic categories. For instance, misclassified faces tend to be labeled as cats (both living objects), whereas misclassified non-living objects tend to be labeled as other non-living objects. This indicates that there are shared representations for images from the same general category.

*Time classification* (Figure 4B). Although all ROIs and time points yield performances above the chance level of 12.5%, the ventral temporal area (which comprises both face and house responsive voxels) yields the best performance. For the latter, classification performance peaks at about 5 s after stimulus onset.

*Searchlight analysis* (Figure 4C). AUC values averaged across subjects are depicted. The AUCs are masked by the significant cluster (*p* < 0.01) and overlayed on an averaged anatomical MRI. Although the cluster is large, high values >0.8 are predominantly found in dorsal and ventral visual areas including the paraphippocampal place area and the fusiform area, nicely dovetailing with the original findings of Haxby et al. (2001).

#### 2.15.3. Benchmarking

**Figure 5:**
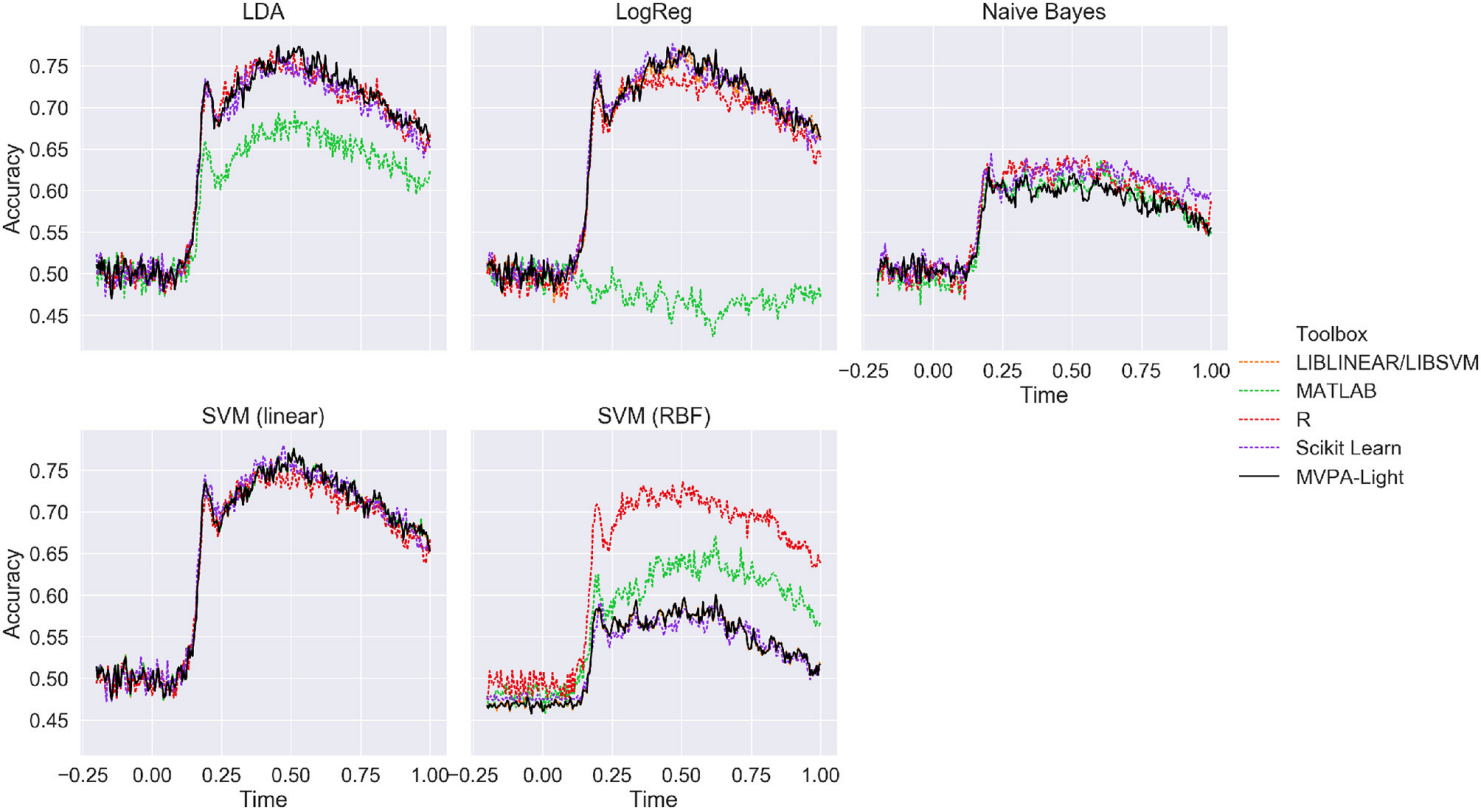
Mean ERP classification accuracy for the benchmarking analysis using the MEG single-subjects data (averaged across subjects). MVPA-Light is depicted as a solid black line.

Figure 5 depicts ERP classification accuracy across time on the MEG single-subjects data for different classifiers and different toolboxes, averaged across subjects. Except for the MATLAB classifiers, results are nearly identical for all implementations of LDA, LogReg, and linear SVM, with a peak performance of about 75%. Lower performance is evident for Naive Bayes, but consistently so across different implementations. For SVM with a RBF kernel, the best performance is obtained in R, followed MATLAB, with both MVPA-Light and Scikit Learn performing worse. Since no hyperparameter tuning was performed, the latter result is most likely due to differences in the default hyperparameters.

Tables 2, 3 show the timing results for different classifiers and regression models. These results are discussed model by model:

**Table 2:** Benchmarking results: mean training time and standard deviation in seconds for different classifiers.

**Dataset**	**Toolbox**	**Classifier**				
		**LDA**	**LogReg**	**Naive Bayes**	**SVM (linear)**	**SVM (RBF)**
MEG single-subjects	MVPA-Light	**0.003** ± 0.0001	**0.0097** ± 0.0005	**0.001** ± 0.00004	0.07 ± 0.002	**0.02** ± 0.0001
	LIBLINEAR	–	0.014 ± 0.0009(*p*) 0.035 ± 0.001(*d*)	–	**0.023** ± 0.002(*p*) 0.231 ± 0.02(*d*)	–
	LIBSVM	–	–	–	0.098 ± 0.01	0.125 ± 0.001
	MATLAB	0.026 ± 0.0008	0.03 ± 0.006	0.05 ± 0.0001	0.041 ± 0.004	0.023 ± 0.0004
	Scikit Learn	0.097 ± 0.0006	0.1 ± 0.005	0.007 ± 0.0001	0.37 ± 0.052	0.45 ± 0.032
	R	0.084 ± 0.0003	0.013 ± 0.002	0.04 ± 0.0001	0.71 ± 0.113	0.41 ± 0.026
MEG super-subject	MVPA-Light	**0.026** ± 0.0028	0.437 ± 0.0062	**0.015** ± 0.0001	10.122 ± 1.05	**5.369** ± 0.033
	LIBLINEAR	–	0.732 ± 0.068(*p*) 0.998 ± 0.063(*d*)	–	**1.338** ± 0.168(*p*) 6.29 ± 0.519(*d*)	–
	LIBSVM	–	–	–	42.089 ± 4.188	37.941 ± 0.404
	MATLAB	0.149 ± 0.002	0.279 ± 0.137	0.231 ± 0.027	20.98 ± 1.78	11.65 ± 0.217
	Scikit Learn	0.596 ± 0.017	2.065 ± 0.109	0.09 ± 0.001	32.19 ± 2.07	34.56 ± 0.38
	R	0.84 ± .004	**0.159** ± 0.018	0.144 ± .0006	1123.16 ± 27.39	123.31 ± 9.38
fMRI	MVPA-Light	**0.293** ± 0.0078	OOM	**0.309** ± 0.011	**0.182** ± 0.0086	**2.064** ± 0.235
	LIBLINEAR	–	**4.008** ± 0.627(*p*) 6.689 ± 1.018(*d*)	-	2.235 ± 0.218(*p*) 6.125 ± 0.995(*d*)	-
	LIBSVM	–	–	–	11.79 ± 0.787	11.88 ± 0.822
	MATLAB	OOM	23.79 ± 4.008	357.49 ± 2.205	5.053 ± 0.325	4.845 ± 0.308
	Scikit Learn	24.45 ± 1.1	20.68 ± 4.24	2.86 ± 0.06	10.46 ± 0.59	9.15 ± 0.59
	R	OOM	7.1 ± 1.13	18.48 ± 0.35	39.67 ± 1.98	43.3 ± 2.18

*For each combination of dataset and classifier, the fastest model is marked in bold. OOM, out of memory error; (p), primal form; (d), dual form*.

**Table 3:** Benchmarking results: mean training time and standard deviation in seconds for different regression models.

**Dataset**	**Toolbox**	**Regression model**			
		**Ridge**	**Kernel Ridge**	**SVR (linear)**	**SVR (RBF)**
MEG single-subjects	MVPA-Light	**0.0016** ± 0.00006	**0.019** ± 0.0001	–	–
	LIBSVM	–	–	0.02 ± 0.001	**0.0041** ± 0.0002
	MATLAB	0.0061 ± 0.0002	–	**0.018** ± 0.037	0.023 ± 0.0005
	Scikit Learn	0.0069 ± 0.0003	0.023 ± 0.003	0.654 ± 0.0647	0.481 ± 0.02
	R	0.055 ± 0.0027	–	1.59 ± 0.094	0.43 ± 0.002
MEG super-subject	MVPA-Light	**0.015** ± 0.001	**7.38** ± 0.023	–	–
	LIBSVM	–	–	**0.653** ± 0.038	**0.121** ± 0.014
	MATLAB	0.186 ± 0.007	–	6.931 ± 0.237	9.9798 ± 0.239
	Scikit Learn	0.062 ± 0.005	14.51 ± 0.21	3.213 ± 0.394	31.61 ± 1.51
	R	0.547 ± 0.0079	–	465.08 ± 49.83	151.66 ± 26.76
fMRI	MVPA-Light	**0.165** ± 0.0042	2.026 ± 0.256	–	–
	LIBSVM	–	–	**4.334** ± 1.48	**2.819** ± 0.0412
	MATLAB	OOM	–	4.545 ± 0.353	4.563 ± 0.284
	Scikit Learn	0.638 ± 0.022	**0.476** ± 0.01	16.138 ± 3.64	9.999 ± 0.59
	R	7.503 ± 0.593	–	37.211 ± 2.056	41.037 ± 2.298

*For each combination of dataset and model, the fastest model is marked in bold. OOM, out of memory error; (p), primal form; (d), dual form*.

*LDA*. The MVPA-Light implementation consistently outperforms other implementations in terms of training time, in some cases by orders of magnitude. For the fMRI dataset, it is almost 100 times faster than Scikit Learn, whereas MATLAB and R both run out of memory. It is worth noting that a shrinkage value of 0.01 was applied for the MVPA-Light and MATLAB implementations. For R, low performance was achieved with rda (regularized LDA), so the standard unregularized LDA was used. For Scikit Learn, the default solver does not allow for shrinkage so no shrinkage was applied.

*LogReg*. The MVPA-Light implementation of Logistic Regression outperforms the competitors for the MEG single-subjects data. It is outperformed by the R implementation for the MEG super-subject. For the fMRI data, it causes an out of memory error and the best performing model is LIBLINEAR.

*Naive Bayes*. The MVPA-Light implementation consistently outperforms other implementations, in some cases by orders of magnitude. Scikit Learn is consistently second best, followed by R and MATLAB.

*SVM*. For linear SVM, LIBLINEAR yields the best training speed except for the fMRI data, where MVPA-Light performs best. For RBF kernels, MVPA-Light's SVM consistently outperforms the competitors, closely followed by MATLAB's fitcsvm. Significant differences are obtained for different toolboxes, with R being the slowest in many cases. The good performance of MVPA-Light's SVM may appear surprising at first glance, given some of its contenders run using C code. First, MVPA-Light uses a large tolerance value; this implies that its algorithm might perform fewer iterations than LIBSVM, although this has not been investigated. If this is the case, it does not seem to be detrimental to classification performance, as Figure 5 illustrates. Second, the advantages of LIBSVM might not play out during a single training iteration. It has an integrated cross-validation procedure, which is likely to be substantially faster than cross-validation using MVPA-Light, although this has not been investigated either.

*Ridge and Kernel Ridge*. MVPA-Light's models lead the field except for the fMRI data, where Scikit Learn's kernel ridge outperforms MVPA-Light. No results are available for R's krr model; it does not appear to have an interface for fixing hyperparameters and instead performs an expensive search using leave-one-out cross-validation, so it was omitted.

*SVR*. MVPA-Light exclusively relies on LIBSVM for SVR, which leads the field except for one case, in which it closely trails the MATLAB implementation. Overall, R yields the slowest implementation.

## 3. Discussion

MVPA-Light offers a suite of classifiers, regression models and metrics for multivariate pattern analysis. A high-level interface facilitates common MVPA tasks such as cross-validated classification across time, generalization, and searchlight analysis. The toolbox supports hyperparameter tuning, pre-computed kernels, and statistical significance testing of the MVPA results.

MVPA-Light also provides a nested preprocessing pipeline that applies operations to training and test sets separately. Among others, it features over- and undersampling, PCA, and scaling operations. It also includes an averaging approach wherein samples are assigned to groups and then averaged in order to increase signal-to-noise ratio. For linear classifiers, this approach has been explored by (Cichy et al., 2015; Cichy and Pantazis, 2017). Recently, it has been generalized to non-linear kernel methods (Treder, 2018). Either approach can be used in the toolbox by adding the operation average_samples or average_kernel to the preprocessing pipeline. To showcase some of its features, analyses of an MEEG (Wakeman and Henson, 2015) and an fMRI (Haxby et al., 2001) dataset are reported. The results illustrate some ways in which the toolbox can aid in quantifying the similarity of representations, measuring the information content, localizing discriminative information in the time-frequency plane, highlighting shared representations across different time points or frequencies, and establishing statistical significance.

A benchmarking analysis was conducted in order to compare MVPA-Light (including LIBSVM and LIBLINEAR) to models provided in the MATLAB Statistics Toolbox, various R packages, and Scikit Learn for Python. While classification performance is largely consistent across different platforms, training time varies considerably. The MVPA-Light implementations of LDA, Naive Bayes, and Ridge Regression consistently outperform their competitors, in some cases by orders of magnitude. For Logistic Regression and SVM, the MVPA-Light implementations and LIBLINEAR lead the field. In all but one case, MVPA-Light's classifiers are faster than the contenders in MATLAB, R, and Scikit Learn. Overall, the fastest classifier is MVPA-Light's LDA and the fastest regression model is MVPA-Light's Ridge Regression. Partially, the success of MVPA-Light is due to specialization: MVPA-Light models tend to have fewer hyperparameters than other models, and MVPA-Light features separate optimized implementations for binary LDA and multi-class LDA, whereas the other toolboxes have a single implementation. Furthermore, MVPA-Light's LDA and Ridge Regression dynamically switch between primal and dual form. This can increase computational efficiency especially when dealing with a large dataset.

The benchmarking results should not be interpreted as final verdicts on the respective toolboxes. Undoubtedly, training speed can be improved by finding an optimal set of hyperparameters for a model. For instance, increasing regularization tends to lead to smoother loss surfaces and often faster convergence for gradient descent algorithms. The strategy for the present analysis was to change default parameters minimally and, if so, only in order to increase comparability e.g., by setting a regularization parameter to a common value. Although MVPA-Light will likely perform well in other situations, too, the present results are mostly indicative of *default performance*, obtained with minimal user interference. This is a relevant measure since it is our belief that the burden of hyperparameter selection should be taken off the user as much as possible.

### 3.1. Setting Up a MVPA Pipeline

If one is spoilt for choice, selecting a model, metrics, and preprocessing steps can be challenging. This section offers practical advice in this regard. Such recommendations tend to be subjective to some extent, hence users are encouraged to perform their own MVPA experiments and compare different models, hyperparameter settings etc. To prevent a statistical bias, extensive experiments should not be performed on the dataset at hand. Instead, a similar dataset e.g., recorded using the same hardware with a similar paradigm can be used for experimentation.

### 3.2. Preprocessing the Data

Although MVPA can be applied to raw data, this may negatively affect performance, so data has ideally been cleaned and corrupted trials have been rejected. It is useful to normalize the data for numerical stability by e.g., z-scoring across trials such that each feature has mean = 0 and standard deviation = 1. This is particularly important for Logistic Regression which uses the exponential function. It also applies to LDA and kernel methods because lack of normalization can lead to results being dominated by the features with the largest scaling. Generally speaking, preprocessing operations should be nested in the cross-validation loop i.e., performed on the training set first and then applied to the test set. The cfg.preprocess option serves this purpose. In some cases such as demeaning, it may be admissible to perform the operation globally on the whole dataset, but one then needs to assure that there is no information leakage from the test set that could bias the results. The same argumentation applies to unsupervised techniques such as PCA. Any preprocessing steps involving the class labels, such as CSP (Blankertz et al., 2008), also need to be nested. Furthermore, for kernel methods, computation can be speeded up by precomputing the kernel matrix using compute_kernel_matrix, although this approach does not work when generalization is required.

### 3.3. Choosing a Classifier

Linear classifiers perform well in a large variety of tasks. LDA is a good default model, since it is fast and robust thanks to regularization (Blankertz et al., 2011). Logistic Regression and linear SVM are more resilient to outliers than LDA, so may be preferred for noisy or strongly non-Gaussian data. Logistic Regression has a hyperparameter-free regularization by default, hence it is more user-friendly than SVM which requires setting the hyperparameter *c*. Naive Bayes should only be used after the features have been decorrelated using PCA or ICA. For non-linear problems, kernel FDA or SVM can be used. Again, SVM requires *c* to be set, whereas for kernel FDA the default regularization often works well. Regarding the choice of a kernel, the RBF kernel is adequate for most classification tasks, but its hyperparameter gamma determining the kernel width might require tuning. If maximizing classification accuracy is vital, it is worth to try an ensemble of classifiers.

### 3.4. Choosing a Regression Model

Ridge regression tends to perform well on a variety of tasks. If the data is noisy, linear Support Vector Regression (SVR) using LIBLINEAR can be applied. If the problem is non-linear, either kernel ridge or kernel SVR using LIBSVM with a RBF kernel is recommended.

### 3.5. Metrics

The most common classification metric is accuracy. For multi-class problems, it is useful to complement it with a confusion matrix. For two classes, AUC is a good alternative to accuracy since it is more robust to class imbalances and invariant to shifts of the classifier threshold. When the roles of the classes are asymmetric (e.g., patients vs. controls), it is useful to report precision and recall along with their harmonic mean (F1 score). If in doubt, report multiple metrics.

### 3.6. Cross-Validation

Classification and regression metrics should be cross-validated. Unless the number of samples is very small, leave-one-out cross-validation should be avoided because it suffers from a large bias; instead, use 5- or 10-fold cross-validation (James et al., 2013). Since samples are randomly assigned to folds, repeating the cross-validation is recommended to get a more stable estimate.

### 3.7. Conclusion

MVPA-Light is a comprehensive toolbox for multivariate pattern analysis. Its models perform competitively compared to other implementations. Future development of MVPA-Light will include additional feature extraction techniques for oscillations, such as Common Spatial Patterns (Blankertz et al., 2008) and the Riemannian geometry approach (Barachant et al., 2013), and further computational improvements, such as efficient permutation testing for LDA/KFDA (Treder, 2019) and faster calculation of the regularization path for SVM (Hastie et al., 2004).

## Data Availability Statement

The MEEG dataset can be found in the OpenNeuro repository (https://openneuro.org/datasets/ds000117/versions/1.0.3). The fMRI dataset can be found on the PyMVPA website (http://www.pymvpa.org/datadb/haxby2001.html). Scripts and figures used in this paper are available in the accompanying GitHub repository (github.com/treder/MVPA-Light-Paper).

## Author Contributions

MT developed the toolbox, performed all analyses and authored the manuscript.

## Conflict of Interest

The author declares that the research was conducted in the absence of any commercial or financial relationships that could be construed as a potential conflict of interest.

## Footnotes

^1^http://www.fieldtriptoolbox.org/tutorial/mvpa_light/

^2^https://github.com/treder/MVPA-Light-Paper
