# Supplementary material for: MVPA-Light: A Classification and Regression Toolbox for Multi-Dimensional Data
Source: Front Neurosci. 2020 Jun 4;14:289. doi: 10.3389/fnins.2020.00289 (PMC7287158; doi:10.3389/fnins.2020.00289)
Supplement: Supplementary file 1 [file Related_Article_1.pdf]

4 APPENDIX

4.1 LDA implementation

The implementation of LDA in MVPA-Light differs from the generative model presented in section 2.4.3. (main paper). It uses the more efficient Fisher Discriminant Analysis (FDA) (Fisher, 1936) which omits calculating probabilities and operates in two steps. In the first step, the data is mapped onto a $(c-1)$-dimensional subspace, where c is the number of classes. In the second step, a sample is assigned to the class with the closest class centroid. LDA thus acts as a prototype classifier within this subspace. The coordinates for the mapping are found by iteratively solving the equation

$$\mathbf{w}_{\text{lda}} = \arg\max_{\mathbf{w}} \frac{\mathbf{w}^\top \mathbf{S}_b \mathbf{w}}{\mathbf{w}^\top \mathbf{S}_w \mathbf{w}} \tag{1}$$

where $\mathbf{S}_b$ and $\mathbf{S}_w$ are defined as

$$\mathbf{S}_b = \sum_{j \in \{1,2,\ldots,c\}} n_j \, (\mathbf{m}_j - \overline{\mathbf{m}})(\mathbf{m}_j - \overline{\mathbf{m}})^\top \qquad \text{(between-classes scatter)}$$

$$\mathbf{S}_w = \sum_{j \in \{1,2,\ldots,c\}} \sum_{i \in \mathcal{C}_j} (\mathbf{x}_i - \mathbf{m}_j)(\mathbf{x}_i - \mathbf{m}_j)^\top \qquad \text{(within-class scatter)}$$

Here, n_j is the number of instances in class j, $\mathbf{m}_j$ is the j-th class mean, $\overline{\mathbf{m}}$ is the sample mean, and $\mathcal{C}_j$ is the set of indices of instances in class j. Note that $\mathbf{S}_w$ is simply the un-normalized version of $\mathbf{\Sigma}$ in Equation 3 (main paper). For two classes, LDA has the simple solution

$$\mathbf{w}_{\text{lda}} = \mathbf{S}_w^{-1} \, (\mathbf{m}_1 - \mathbf{m}_2). \tag{2}$$

For more than two classes, there is multiple vectors $\mathbf{w}_{\text{lda}}$. They are collected in a matrix $\mathbf{W} \in \mathbb{R}^{p \times (c-1)}$ and scaled such that $\mathbf{W}^\top \mathbf{S}_w \mathbf{W} = \mathbf{I}$ (Bishop, 2007). $\mathbf{W}$ can be obtained via the generalized eigenvalue problem $\mathbf{S}_b \, \mathbf{W} = \mathbf{S}_w \, \mathbf{W} \mathbf{\Lambda}$ where $\mathbf{\Lambda}$ is a diagonal matrix of eigenvalues. The threshold is calculated under the assumption of equal probabilities for the classes. $\mathbf{S}_w$ is often ill-conditioned or singular and hence the inverse in Equation 2 cannot be calculated reliably. Therefore, shrinkage regularization is applied by default and $\mathbf{S}_w$ is replaced by $\widetilde{\mathbf{S}}_w$:

$$\widetilde{\mathbf{S}}_w = (1 - \lambda) \, \mathbf{S}_w + \lambda \, \nu \, \mathbf{I} \tag{3}$$

where $\mathbf{I}$ is the identity matrix and $\nu = \text{trace}(\mathbf{S}_w)/p$ (Blankertz et al., 2011). For $\lambda = 1$, LDA becomes a prototype classifier, that is, each sample is assigned to the closest centroid in input space. The effect of shrinkage regularization is depicted in Figure 1. In the literature, LDA has also been used with ridge-regression type regularization, yielding $\widetilde{\mathbf{S}}_w = \mathbf{S}_w + \lambda_{\text{ridge}} \, \mathbf{I}$ with $\lambda_{\text{ridge}} \in [0, \infty)$ (Friedman, 1989). The user can switch to ridge regularization by setting `reg = 'ridge'`. However, both regularization approaches are equivalent up to scaling. For a given shrinkage value $\lambda < 1$, the corresponding ridge regularization value is $\lambda_{\text{ridge}} = \nu\lambda/(1-\lambda)$.

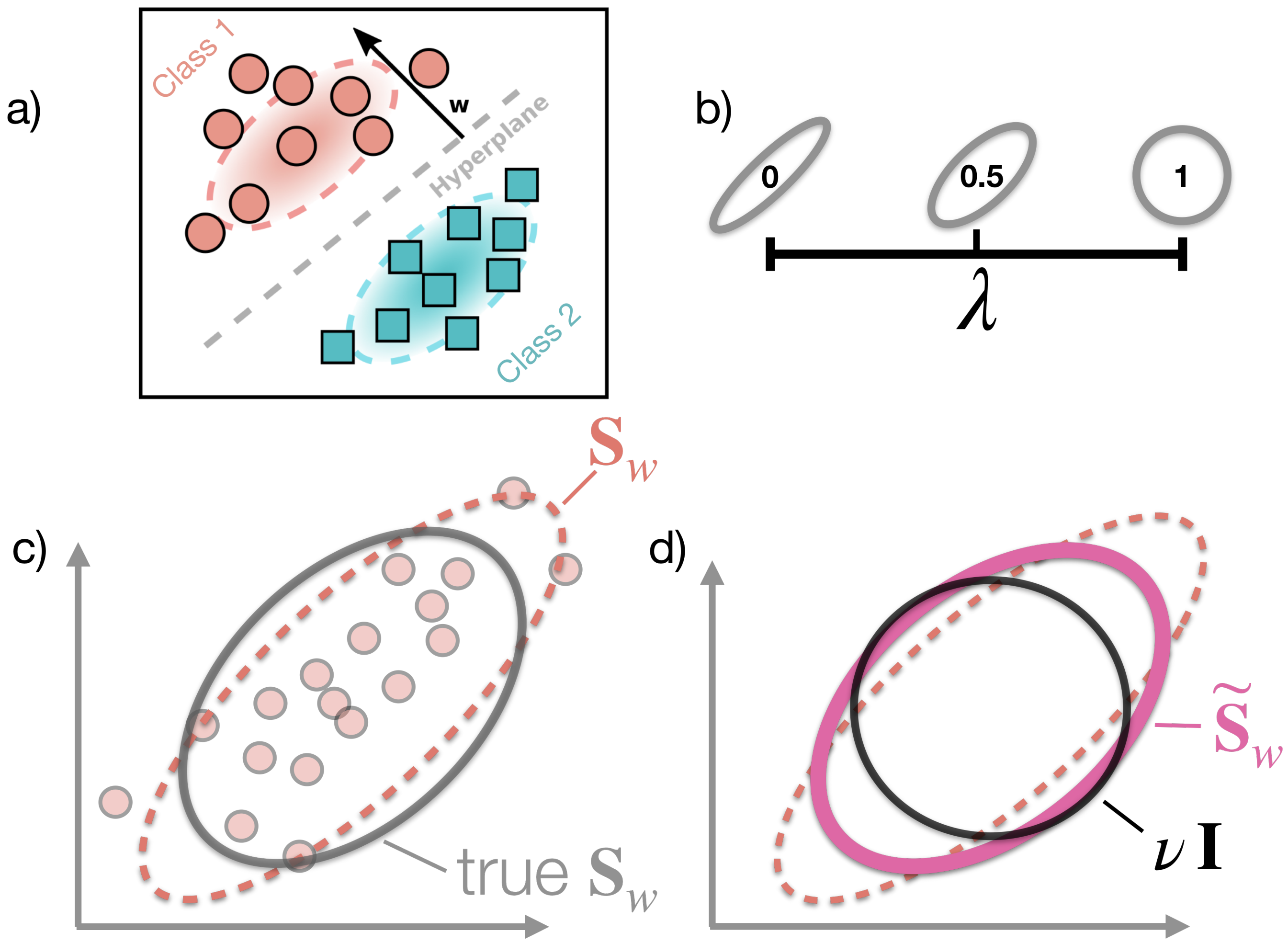

Figure 1. a) LDA with two classes. Covariance matrices are indicated by ellipses. The weight vector $\mathbf{w}$ is the normal to the hyperplane. b) Effect of varying λ on the shape of the covariance matrix. c) Covariance matrices estimated from data usually overestimate large eigenvalues (Blankertz et al., 2011). d) The shrinkage estimate $\widetilde{\mathbf{S}}_w$ partially corrects the estimation error by shrinking towards a spherical covariance $\nu\mathbf{I}$.

If $p \gg n$, that is, the number of features is much larger than the number of samples, calculation and inversion of the [features $\times$ features] covariance matrix can be computationally expensive. In these cases, the [samples $\times$ samples] Gram matrix can be used instead. The hyperparameter `form` decides on whether the 'primal' form based on covariance or 'dual' form based on the Gram matrix is used. By default (`form = 'auto'`), this is decided automatically by comparing n and p. For the dual form, the resultant formula is equivalent to kernel FDA with a linear kernel. However, the regularization of $\mathbf{N}$ in kernel FDA is not equivalent to the regularization of $\mathbf{S}_w$. Furthermore, to the best of our knowledge, the Ledoit-Wolf estimate has not been reported in the literature using dual notation. Therefore, a dual regularization approach for LDA is developed next.

4.1.1 Dual regularization of LDA

The goal of this section is to develop a dual regularization approach for LDA that is equivalent to the primal regularization approach. This is important in order to assure that switching from primal to dual form with an equal regularization magnitude λ does not affect the solution $\mathbf{w}$. The approach consists of

two steps. First, the Ledoit-Wolf estimate for the optimal regularization hyperparameter λ^* needs to be calculated using the Gram matrix instead of the covariance matrix. Second, it needs to be assured that the dual regularization yields the same $\mathbf{w}$ as the primal approach.

4.1.2 Dual Ledoit-Wolf estimate

The Ledoit-Wolf estimate (Ledoit and Wolf, 2004; Blankertz et al., 2011) for the optimal regularization hyperparameter, denoted as λ^*, can be formulated as

$$\lambda^* = \frac{\sum_{i=1}^{n} ||\mathbf{S} - \mathbf{x}_i\mathbf{x}_i^\top||_F^2}{n^2[\,\mathrm{Tr}(\mathbf{S}^2) - \mathrm{Tr}(\mathbf{S})^2/p\,]} \tag{4}$$

where $\mathbf{S} = \frac{1}{n}\mathbf{X}^\top\mathbf{X}$, Tr is the trace operator and $\mathbf{X}$ is assumed to be zero mean (column-wise). In a finite sample, λ^* can take values smaller than 0 or larger than 1, so it is additionally thresholded between 0 and 1.

To rewrite Equation 4 in terms of the Gram matrix $\mathbf{K} = \mathbf{X}\mathbf{X}^\top$ observe that in the numerator we can write $||\mathbf{S} - \mathbf{x}_i\mathbf{x}_i^\top||_F^2 = \mathrm{Tr}(\mathbf{K}^2)/n^2 - 2\,\mathrm{Tr}(\mathbf{S}\,\mathbf{x}_i\mathbf{x}_i^\top) + \langle\mathbf{x}_i, \mathbf{x}_i\rangle^2$. For the middle term we have $\mathrm{Tr}(\mathbf{S}\,\mathbf{x}_i\mathbf{x}_i^\top) = \mathrm{Tr}(\frac{1}{n}\sum_{j=1}^{n}\mathbf{x}_j\mathbf{x}_i^\top\langle\mathbf{x}_i, \mathbf{x}_j\rangle) = \frac{1}{n}\sum_{j=1}^{n}\langle\mathbf{x}_i, \mathbf{x}_j\rangle^2$. In other words, we can rewrite the Ledoit-Wolf estimate in terms of inner products as

$$\lambda^* = \frac{\mathrm{Tr}(\mathbf{K}^2)/n - 2\sum_{i=1}^{n}\sum_{j=1}^{n}\mathbf{K}_{ij}^2/n + \sum_{i=1}^{n}\mathbf{K}_{ii}^2}{\mathrm{Tr}(\mathbf{K}^2) - \mathrm{Tr}(\mathbf{K})^2/p} \tag{5}$$

where the identity $\mathrm{Tr}(\mathbf{S}^2) = \mathrm{Tr}(\mathbf{K}^2)/n^2$ has been used. Both primal and dual estimation of λ^* are implemented in the function `LedoitWolfEstimate`. This approach generalizes to kernel FDA when the Gram matrix is replaced by the kernel matrix.

4.1.3 Dual regularization

The relationship between primal weights $\mathbf{w}$ and dual weights $\boldsymbol{\alpha}$ is given by $\mathbf{w} = \mathbf{X}^\top\boldsymbol{\alpha}$. The denominator of the Fisher ratio in Equation 1 can be related to the dual problem as $\mathbf{w}^\top\mathbf{S}_w\mathbf{w} = \boldsymbol{\alpha}^\top\mathbf{N}\boldsymbol{\alpha}$ (Ghojogh et al., 2019) where $\mathbf{N}$ is the "dual" of $\mathbf{S}_w$ (see Equation 12). For the ridge regularized case, we then obtain

$$\mathbf{w}^\top(\mathbf{S}_w + \lambda\mathbf{I})\,\mathbf{w} = \boldsymbol{\alpha}^\top(\mathbf{N} + \lambda\mathbf{K})\boldsymbol{\alpha} \tag{6}$$

where $\mathbf{K}$ is the Gram matrix as defined above. The optimal $\boldsymbol{\alpha}$ is given by $(\mathbf{N}+\lambda\mathbf{K})^{-1}(\mathbf{M}_1 - \mathbf{M}_2)$ where $\mathbf{M}_j$ is defined as in Equation 12. The result for shrinkage regularization is analogous.

4.2 Naive Bayes implementation

MVPA-Light uses Gaussian distributions to model the univariate densities, that is $P(x^{(j)}\,|\,y = i) = \frac{1}{\sqrt{2\pi\sigma_{ij}^2}}\exp(-\frac{(x^{(j)} - m_{ij})^2}{2\sigma_{ij}^2})$. The parameters of the model are thus the m_{ij}'s (mean of the j-th feature in class i) and σ_{ij}^2's (variance of the j-th feature in class i) estimated on the training data. Inserting the Gaussian densities into Equation 2 (main paper) yields

$$P(y=i\,|\,\mathbf{x}) = \frac{\prod_{j=1}^{p} \frac{1}{\sqrt{2\pi\sigma_{ij}^2}} \exp(-\frac{(x^{(j)}-m_{ij})^2}{2\sigma_{ij}^2})\; P(y=i)}{\sum_{k=1}^{c} \prod_{j=1}^{p} \frac{1}{\sqrt{2\pi\sigma_{kj}^2}} \exp(-\frac{(x^{(j)}-m_{kj})^2}{2\sigma_{kj}^2})\; P(y=k)}. \tag{7}$$

Classification is computationally more efficient if the denominator (which is necessary for normalization only) is omitted and the numerator is log transformed. This leads to the class-conditional decision values d_i,

$$d_i = -\frac{1}{2}\sum_{j=1}^{p} \log(2\pi\,\sigma_{ij}^2) - \sum_{j=1}^{p} \frac{(x^{(j)}-m_{ij})^2}{2\sigma_{ij}^2} + \log P(y=i) \tag{8}$$

which can be transformed into posterior probabilities using the softmax function

$$P(y=i\,|\,\mathbf{x}) = \frac{e^{d_i}}{\sum_{k=1}^{c} e^{d_k}}. \tag{9}$$

4.3 Logistic Regression implementation

The optimization problem underlying Logistic Regression is convex but there exists no analytical solution. Instead, an iterative algorithm is required to optimize $\mathbf{w}$. In MVPA-Light, the Trust Region Newton Method introduced by Lin et al. (2007) is implemented in the function `TrustRegionDoglegGN`. Log-F(1,1) regularization (`reg = 'logf'`) is implemented via data augmentation. L2-regularization is implemented by adding a penalty term to the loss function:

$$\mathcal{L}_{\text{LR}}^{L2}(\mathbf{w}) = \mathcal{L}_{\text{LR}}(\mathbf{w}) + \frac{\lambda}{2}\,||\mathbf{w}||^2 \tag{10}$$

Here, $\lambda \in [0,\infty)$ controls the amount of regularization. While log-F(1,1) regularization does not require any hyperparameters, L2-regularization requires λ to be set. The effect of regularization on the predicted probabilities is depicted in Figure 2. If multiple candidates are provided for λ, a line search is performed using nested cross-validation. This kind of line search is costly, since the classifier has to be trained multiple times for each value of the hyperparameter. To speed up the search, 'warm starts' can be used wherein the initial value for $\mathbf{w}$, denoted as $\mathbf{w}_{\text{init}}$, is a function of the solutions in previous iterations. If `predict_regularization_path=1`, then in the k-th iteration, a polynomial function is fit to the solutions $\mathbf{w}_{k-1}, \mathbf{w}_{k-2}, ...$ to the previous iterations using $\lambda_{k-1}, \lambda_{k-2}, ...$ as predictors. It is then evaluated at the current value of λ in order to predict a good starting vector for the optimization.

4.4 SVM implementation

The formulation of SVM in Equation 6 (main paper) is intuitive but limited to the linear case. It can be rewritten into a dual formulation which applies to both linear and kernel case (Hsieh et al., 2008). The optimal weights are then found by solving the quadratic optimization problem

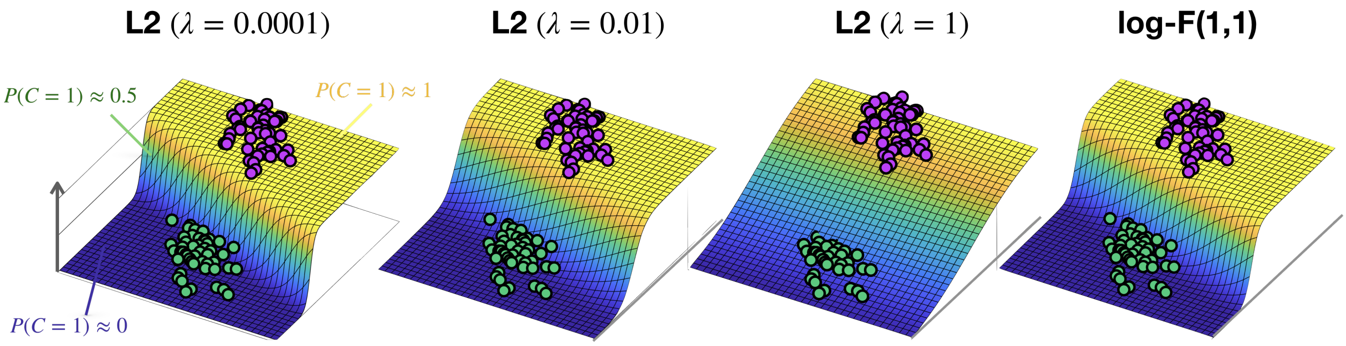

Figure 2. Logistic regression on data with two features (x and y axis) and two classes represented by purple (class 1) and green (class 2) dots. The curved surface is the sigmoid function fit and the vertical z-axis represents the probability for class 1. In L2 regularization, a larger λ leads to a flatter sigmoid function with smoothly varying probabilities. For comparison, the sigmoid fit using log-F(1,1) regularization is also shown.

$$\begin{aligned} \underset{\boldsymbol{\alpha}}{\arg\min} \quad & \frac{1}{2}\boldsymbol{\alpha}^\top \mathbf{Q}\,\boldsymbol{\alpha} - \mathbb{1}^\top \boldsymbol{\alpha} \\ \text{subject to} \quad & \forall i : 0 \leq \alpha_i \leq c \end{aligned} \tag{11}$$

where $\boldsymbol{\alpha} = [\alpha_1, \alpha_2, ..., \alpha_n]^\top \in \mathbb{R}^n$ is the dual weight vector and $\mathbb{1}$ is a vector of 1's. $\mathbf{Q}$ is the kernel matrix with the class labels absorbed, i.e. $\mathbf{Q}_{ij} = y_i y_j\, k(\mathbf{x}_i, \mathbf{x}_j)$, where k is the kernel function, and $y_i, y_j \in \{+1, -1\}$ are the class labels for the i-th and j-th sample. The hyperparameter `c` controls the amount of regularization. The optimization of the weights is performed using a Dual Coordinate Descent approach (Hsieh et al., 2008) implemented in the function `DualCoordinateDescent`.

4.5 KFDA implementation

Let $\mathbf{K} \in \mathbb{R}^{n\times n}$ be the kernel matrix representing the inner products of the samples in the Reproducing Kernel Hilbert Space (RKHS) and $\mathbf{K}_j \in \mathbb{R}^{n\times n_j}$ be the submatrix of $\mathbf{K}$ with columns corresponding to samples in class j. The kernelized versions of between-classes and within-class scatter are given by the matrices $\mathbf{M}$ and $\mathbf{N}$ as

$$\begin{aligned} \mathbf{M} &= \sum_{j=1}^{c} n_j\, (\mathbf{M}_j - \mathbf{M}_*)\, (\mathbf{M}_j - \mathbf{M}_*)^\top \\ \mathbf{N} &= \sum_{j=1}^{c} \mathbf{K}_j\, (\mathbf{I} - \frac{1}{n_j} \mathbb{1}\, \mathbb{1}^\top)\, \mathbf{K}_j^\top \end{aligned} \tag{12}$$

where $\mathbf{M}_j \in \mathbb{R}^n$ is the mean of the columns of $\mathbf{K}_j$ and $\mathbf{M}_*$ is the mean of the columns of $\mathbf{K}$. Then the solution is given by the $(c-1)$ leading eigenvectors of $\mathbf{N}^{-1}\mathbf{M}$. Like in LDA, the model can be regularized using ridge regularization (`reg = 'ridge'`) or shrinkage regularization (`reg = 'shrink'`). Using shrinkage, $\mathbf{N}$ is replaced by $\widetilde{\mathbf{N}} = (1-\lambda)\, \mathbf{N} + \lambda\, \mathrm{trace}(\mathbf{N})/n\, \mathbf{I}$.

4.6 Implementing a prototype classifier

This section illustrates how to implement a prototype classifier that assigns a new sample to the class with the closest class centroid. Class centroids are calculated from the training data by calculating the means of the samples within each class. This example is mostly didactic. A prototype classifier can be simulated with LDA or multi-class LDA by setting `reg = 'shrink'` and `lambda = 1`.

First, the train function needs to be implemented. As input arguments, train functions take a `param` struct which is short notation for 'hyperparameter'. It corresponds to the `cfg.hyperparameter` struct defined in the high-level functions. Additionally, it takes the training data `X` and the corresponding class labels `clabel`. For brevity, most of the documentation is omitted.

```
function model = train_prototype(param, X, clabel)

nclasses = max(clabel);

% Classifier struct
model  = [];
model.nclasses = nclasses;

%% Calculate class centroids
model.centroid = zeros(nclasses, size(X,2));
for c=1:nclasses
    model.centroid(c,:) = mean(X(clabel==c,:));
end
```

The output of the train function is a structure `model` that describes the parameters of the classifier after training. The test function takes the classifier `model` and the test data `X` as input. For classification, we need to calculate the Euclidean distance between each test sample and each of the centroids. Then, each sample is assigned to the closest class centroid.

```
function clabel = test_prototype(model, X)

% Euclidean distance of each sample
% to each class centroid
dist = arrayfun( @(c) sum( bsxfun(@minus, X, ...
    model.centroid(c,:)).^2, 2), ...
    1:model.nclasses, 'Un',0);
dist = cat(2, dist{:});

% For each sample, find the closest centroid
clabel = zeros(size(X,1),1);
for ii=1:size(X,1)
    [~, clabel(ii)] = min(dist(ii,:));
end
```

The output of the test function is `clabel`, the vector of predicted class labels. Finally, an entry for `prototype` needs to be added to the function `mv_get_hyperparameter`. Since the prototype classifier has no hyperparamaters, this entry can be empty. Provided that the train and test functions are in the MATLAB path, the new classifier can be used with all high-level functions by setting `cfg.model = 'prototype'`.

REFERENCES

Bishop, C. M. (2007). Pattern Recognition and Machine Learning. *Journal of Electronic Imaging* 16, 049901. doi:10.1117/1.2819119

Blankertz, B., Lemm, S., Treder, M., Haufe, S., and Müller, K. R. (2011). Single-trial analysis and classification of ERP components - A tutorial. *NeuroImage* 56, 814–825. doi:10.1016/j.neuroimage.2010.06.048

Fisher, R. A. (1936). The use of multiple measurements in taxonomic problems. *Annals of Eugenics* 7, 179–188. doi:10.1111/j.1469-1809.1936.tb02137.x

Friedman, J. H. (1989). Regularized Discriminant Analysis. *Journal of the American Statistical Association* 84, 165. doi:10.2307/2289860

Ghojogh, B., Karray, F., and Crowley, M. (2019). Fisher and Kernel Fisher Discriminant Analysis: Tutorial. *arXiv e-prints* , arXiv:1906.09436

Hsieh, C.-J., Chang, K.-W., Lin, C.-J., Keerthi, S. S., and Sundararajan, S. (2008). A dual coordinate descent method for large-scale linear SVM. In *Proceedings of the 25th international conference on Machine learning - ICML '08* (New York, New York, USA: ACM Press), 408–415. doi:10.1145/1390156.1390208

Ledoit, O. and Wolf, M. (2004). A well-conditioned estimator for large-dimensional covariance matrices. *Journal of Multivariate Analysis* 88, 365–411. doi:10.1016/S0047-259X(03)00096-4

Lin, C.-J., Weng, R. C., and Keerthi, S. S. (2007). Trust region Newton methods for large-scale logistic regression. In *Proceedings of the 24th international conference on Machine learning - ICML '07* (New York, New York, USA: ACM Press), 561–568. doi:10.1145/1273496.1273567
